# Lipidomic chemotaxonomy aligned with phylogeny of Halobacteria

**DOI:** 10.3389/fmicb.2023.1297600

**Published:** 2023-11-24

**Authors:** Wenyong Yao, Wan Zhang, Wei He, Wenjie Xiao, Yufei Chen, Yuanqing Zhu, Fengfeng Zheng, Chuanlun Zhang

**Affiliations:** ^1^Shenzhen Key Laboratory of Marine Archaea Geo-Omics, Department of Ocean Science and Engineering, Southern University of Science and Technology, Shenzhen, China; ^2^GEOMAR Centre for Marine Biotechnology (GEOMAR-Biotech), Research Unit Marine Natural Products Chemistry, GEOMAR Helmholtz Centre for Ocean Research Kiel, Kiel, Germany; ^3^Department of Biology, Hadal, Nordcee & DIAS, University of Southern Denmark, Odense, Denmark; ^4^Shanghai Sheshan National Geophysical Observatory, Shanghai, China

**Keywords:** Halobacteria, lipidomics, phylogeny, chemotaxonomy, network-coordinated features, bioinformatics

## Abstract

Archaea play an important role in global biogeochemical cycles and are considered ancestral to eukaryotes. The unique lipid composition of archaea, characterized by isoprenoid alkyl chains and ether linkage to glycerol-1-phosphate, offers valuable insights into archaeal phylogeny and evolution. However, comprehensive studies focusing on archaeal lipidomes, especially at the intact polar lipid level, are currently limited. Here, we built an in-house library of archaeal lipids by using high-performance liquid chromatography coupled with mass-spectrometry, which was integrated with bioinformatics and molecular network analyses. Seven halobacterial strains, representing three distinct orders, were cultured under identical conditions to investigate their lipidomes. A total of 162 features were identified, corresponding to 107 lipids that could be assigned to different strains. Clustering analyses of both core lipids and total lipids matched the phylogeny of Halobacteria at the order level. Notably, lipids such as triglycosyl diether-phosphatidyl acid and bis-sulfate glycosyl lipids were specific to particular groups and could serve as diagnostic intact lipid biomarkers for Halobacteria. Furthermore, the analysis of network-coordinated features facilitated the linkage of unknown lipid compounds to phylogeny, which promotes a lipidome to phylogeny matchup among three *Haloferax* strains, thereby expanding the knowledge of the halobacterial lipidome. Our study provides a comprehensive view of the lipidomes of the seven strains of Halobacteria and highlights the potential of lipidomics for studying archaeal phylogeny.

## 1 Introduction

The identification of archaea has widely broadened our knowledge of life forms on Earth and motived the proposal of three domain theory (Woese et al., 1990). Archaea are ubiquitously found in a wide variety of geological settings, from extreme conditions such as salt lakes, hot springs, and hydrothermal vents, to more commonplace settings such as soils, lakes, rivers, marine water column and sediments (Baker et al., 2020). Although only a limited number of archaeal taxa have been isolated and cultured, advances in sequence-based approaches such as 16S rRNA and metagenomic sequencing have recovered a high phylogenetic diversity and versatile metabolic potentials of archaea, suggesting they might play an important role in multiple elemental cycling processes (Baker et al., 2020). Recently, the newly discovered Asgard archaea showed a close phylogenetic relationship with eukaryotes, and their genomes encode an expanded repertoire of eukaryotic signature proteins (Spang et al., 2015; Baker et al., 2020), pointing to an archaeal origin of eukaryotes (Zaremba-Niedzwiedzka et al., 2017; MacLeod et al., 2019). These new evidences largely challenged the three domains theory, highlighting the need for further research on archaeal phylogeny and evolution.

Archaea are characterized for their cell membrane lipids consisting of isoprenoid alkyl chains that are ether-linked to glycerol-1-phosphate, whereas bacterial and eukaryotic membranes are mostly comprised of fatty acids that are ester-linked to glycerol-3-phosphate (Valentine, 2007; Koga, 2011). The membrane of archaea is composed of dialkyl glycerol diethers (e.g., archaeol) or glycerol dialkyl glycerol tetraethers (e.g., GDGTs) or both. The structure of ether bound lipids in archaea is more stable than bacterial and eukaryotic counterparts, which is considered as one of the key strategies for archaea to adapt to extreme conditions and prolongs the preservation of fossil archaeal biomarkers (Summons et al., 2022). Compared with DNA, RNA and proteins, lipids do not contain phylogenetic signals, but are suitable biomarkers with longer lifespan and diverse structures to indicate ecological occurrence and activity of archaea (Briggs and Summons, 2014). The distribution of lipids is suggested to be regulated by both genetic constraints and physiological modification responding to environmental stresses. Therefore, archaeal lipids are widely used as biomarkers to reconstruct paleoenvironment and ancient biological activities (reviewed by Schouten et al., 2013). They also serve as useful chemotaxonomic biomarkers as each taxon may produce a distinct lipid profile (e.g., Elling et al., 2017).

Halobacteria (also known as Haloarchaea) is a subgroup of *Euryarchaeota*, consisting of three main orders, namely *Halobacteriales*, *Haloferacales*, and *Natrialbales* (Gupta et al., 2015, 2016). These microorganisms can resist saline stress and survive in diverse hypersaline environments such as salt lakes and salt marshes (Oren, 2014). The membrane lipids of Halobacteria primarily consist of bilayer diethers, with the absence of monolayer tetraether lipids (Villanueva et al., 2014; Bale et al., 2019). The structural diversity of halobacterial archaeol cores can be extended by replacing a C_20_ phytanyl chain with an elongated C_25_ chain and/or by forming several double bonds (uns) on the phytanyl chain (Dawson et al., 2012; Bale et al., 2019; Vandier et al., 2021). This structural flexibility enables them to adapt and survive in hypersaline environments (Dawson et al., 2012). The diether core structures in Halobacteria can be further linked to a phospho- and/or glycosyl-head group, forming intact polar lipids (IPLs). Phosphatidyl glycerol (PG) and methylated phosphatidyl glycerol phosphate (Me-PGP) are the dominant IPL components identified in Halobacteria (Kamekura and Kates, 1999; Sprott et al., 2004; Kellermann et al., 2016). PG and Me-PGP are negatively charged phospholipids that can interact with cations, which helps the organisms to resist osmotic shock in hypersaline environments (Tenchov et al., 2006). The halobacterial lipids also include the cardiolipin analogs (archaeal cardiolipin) that have two core structure of diethers sharing one polar head group (Sturt et al., 2004; Corcelli, 2009). Menaquinone (MK, also known as vitamin K2) that resides inside the bilayer as an electron carrier in the respiratory chains (Kellermann et al., 2016), and carotenoid (mainly bacterioruberin) which is responsible for the red appearance of Halobacteria (Hartmann et al., 1980), are important non-bilayer forming lipids in the Halobacteria.

Hitherto, hundreds of diverse lipid components have been identified in Halobacteria and other archaeal categories using a variety of analytical techniques, such as NMR, GC-MS, LC-MS (Bale et al., 2019; Law and Zhang, 2019). Recent advances in lipidomics with high-resolution mass spectrometry (HRMS) have significantly improved the detection coverage, sensitivity, and throughput. This enables the analysis of a larger number of lipids in a single run (Tsugawa et al., 2020), which have continuously enhanced our knowledge of archaeal lipidome (Knappy et al., 2009; Law et al., 2021). Remarkably, the structural diversity and chemotaxonomic potential have been carefully examined with HRMS-based lipidomic approach for Thaumarchaeota (Elling et al., 2017), halo(alkali)philic methanogens and Halobacteria (Bale et al., 2019). However, the data processing of HRMS is still time-consuming and the identifications largely relied on manual interpretation, which may substantially affect the efficiency and accuracy of archaeal lipids analysis.

The development of bioinformatic tools has facilitated the analysis of the expanded dataset, which can provide reliable lipid identification by integrating multidimensional mass spectrometry information, for instance, MS^1^, retention time (RT) and the MS^2^ spectra (Tsugawa et al., 2020; Schmid et al., 2021). Bioinformatic approaches such as feature-based molecular networking have been widely used in HRMS-based lipidomic or metabolic research, allowing further mass spectra interpretation by linking the features with similar MS^2^ spectra to generate a molecular network (Guthals et al., 2012; Watrous et al., 2012). This approach has increasingly been used in uncovering the lipidomes from environmental and pure culture samples, which enables the visualization of the relationships between different lipid features, the categorization of unknown features, and the structural prediction of unknown analogs (Ding et al., 2021, 2022).

In a specific environmental niche, different archaeal species will encounter the same environmental/growth conditions. This underscores the necessity of conducting a lipidome investigation under controlled conditions. In this study, we hypothesize that the lipid composition and abundance of halobacterial strains are in alignment with the phylogeny, which may manifest the underlying mechanisms governing phylogenetic control over lipid biosynthesis. We cultured seven strains from three prominent halobacterial orders under identical conditions, minimizing the potential confounding effects of environmental fluctuations. We employed and assessed the application of ultra-high performance liquid chromatography-high resolution mass spectrometry (UPLC-HRMS) coupled with bioinformatics tools. This encompassed an *in silico* spectral library-based lipid identification and a feature-based molecular network approach. The streamlined procedure of lipidomic analysis using our in house library allowed us to comprehensively characterize the lipidomic composition of archaea and evaluate their chemotaxonomic potential. This study expands our understanding of the archaeal lipidome, which may provide a new insight for exploring the relevance between lipidome and phylogeny.

## 2 Materials and methods

### 2.1 Cultivation of halobacterial strains

**Figure 1:**
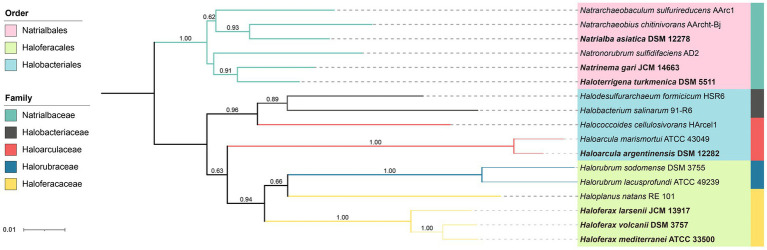
Neighbor-joining phylogenetic tree of Halobacteria based on the 16S rRNA gene. The strains studied are depicted in bold font. 16S rRNA genes of other stains were obtained from NCBI database. The tree was bootstrapped 1,000 times and values above 0.5 are marked. The branch length represents the evolutionary distance between the taxa.

Seven pure cultured strains of Halobacteria were used in this study, including *Haloarcula* (*Ha.*) *argentinensis*; *Haloferax* (*Hf.*) *larsenii*; *Haloferax mediterranei*; *Haloferax volcanii*; *Haloterrigena* (*Ht.*) *turkmenica*; *Natrialba asiatica (Na.)* and *Natrinema (Nt.) gari* (Table 1). *Hf. larsenii*, *Hf. mediterranei* and *Hf. volcanii* are from the order of *Haloferacales*; *Nn. gari*, *Ht. turkmenica* and *Na. asiatica* are from the order of *Natrialbales*, with *Na. asiatica* being distantly related to *Ht. turkmenica* and *Nn. gari*. *Ha. argentinensis* is the only species in the order *Halobacteriales* (Figure 1). All strains were grown at 37°C and 200 rpm in triplicate using 300 mL of DSMZ 589 liquid medium, which was originally designed for cultivating *Halobacterium lacusprofundii*. The medium contained NaCl 180 g/L; MgCl_2_∙6H_2_O 75 g/L; MgSO_4_∙7H_2_O 7.4 g/L; KCl 7.4 g/L; CaCl_2_∙2H_2_O 1 g/L; yeast extract 1 g/L; sodium succinate 10 g/L; vitamin solution 1 mL/L. The growth pH was adjusted to 7.4. The vitamin solution was a mixture of biotin 0.1 g/L; vitamin B_12_ 0.1 g/L; and thiamine-HCl∙2H_2_O 0.1 g/L. Cell growths were monitored at a wavelength of 600 nm (OD600) and harvested at the stationary phase. 45 mL of the cultures were harvested by centrifuging at 10000 g for 12 min; the cell pellets were collected, freezing-dried, and stored at −80°C for lipid extraction.

**Table 1:** Strains used in this study and their optimum growth conditions.

Species	Accession No.	Temperature °C	NaCl M	pH	GC %	References
*Haloarcula argentinensis*	DSM 12282	40	2.5–3.0	7.5	62.0	Ihara et al. (1997)
*Haloferax larsenii*	JCM 13917	43–45	2.2–3.4	6.5–7.0	62.2	Xu et al. (2007)
*Haloferax mediterranei*	ATCC 33500	35–37	3.4	6.5	60.0	Rodriguez-Valera et al. (1983)
*Haloferax volcanii*	DSM 3757	45	1.7–2.5	7.0	63.5	Mullakhanbhai and Larsen (1975)
*Haloterrigena turkmenica*	DSM 5511	45–51	2.6–3.4	7.4	59.8	Minegishi and Kamekura (2009) and Zvyagintseva and Tarasov (1988)
*Natrialba asiatica*	DSM 12278	30–40	4.0	6.6–7.0	62.3	Hezayen et al. (2001)
*Natrinema gari*	JCM 14663	37–40	2.6–3.4	6.0–6.5	65.4	Tapingkae et al. (2008)

### 2.2 Lipid extraction

The cell pellets were lyophilized and extracted using a modified Bligh and Dyer method (Findlay, 2004). Briefly, the samples were resuspended with 3.2 mL of 50 mM phosphate buffer (PB, dissolve 8.7 g K_2_HPO_4_ in 1 L distilled H_2_O, pH was adjusted to 7.4 using HCl) and transferred to Teflon centrifuge tubes (Thermo Scientific). The solvent mixture was adjusted to a ratio of 2:1:0.8 (methanol (MeOH): dichloromethane (DCM): PB, v/v/v) by adding 8 mL methanol, and 4 mL dichloromethane. 1-O-hexadecyl-2-acetyl-sn-glycerol-3-phosphocholine (C_16_-PAF, 0.1 μg/μL) and 1,2-dinonadecanoyl-sn-glycerol-3-phosphocholine (C_19_-PC, 50 pmol/μL) were spiked as internal standards. The tubes were vortexed and then ultrasonicated for 2 min (twice with 1-min intervals). Subsequently, the samples were incubated in dark at the 4°C overnight (Findlay, 2004). The solvent ratio was adjusted to 1:1:0.9 (MeOH: DCM: H_2_O, v/v/v) by adding 4 mL ddH_2_O and 4 mL DCM. The lipid extracts were left to stand briefly for phase separation. The DCM layer was collected, and the remaining aqueous phase was re-extracted twice with 5 mL of DCM. Samples from all DCM layer collection were pooled as total lipid extracts (TLEs), which were dried under a gentle nitrogen flow and stored at −80°C until lipid analysis.

### 2.3 Lipid analyses

The TLEs were dissolved in 1 mL MeOH and 10 μL of each sample was pooled as a QC sample for peak alignment. An aliquot of the TLEs was analyzed on a Waters ACQUITY I-class ultra-performance liquid chromatography (UPLC) coupled to Waters SYNAPT G2-S*i* quadrupole time-of-flight mass spectrometer (qTOF); electrospray ionization (ESI) in positive mode was equipped as the ion source.

Separation of archaeal lipids was achieved on a Waters Acquity BEH C_18_ column maintained at 45°C (Wörmer et al., 2013; Kellermann et al., 2016). The samples were kept at 7°C and the flow rate was kept at 0.3 mL/min during the run. Solvent A was 85% MeOH (Optima™ LC/MS Grade, Fisher Chemical) with 15% H_2_O (Optima™ LC/MS Grade, Fisher Chemical) and solvent B was 50% isopropanol (Optima™ LC/MS Grade, Fisher Chemical) with 50% MeOH, both amended with 0.1% NH_4_OH (25–30% NH_3_ basis, Sigma-Aldrich) and 0.04% formic acid (>99.0%, Optima™ LC/MS Grade, Fisher Chemical). The lipids were eluted with the following gradient: 100% A was maintained for 2.5 min. B was linearly increased to 15% at 3 min, 85% at 27 min, and 100% at 28 min and kept the fraction until 37 min. The flow was reversed to 100% A at 38 min and re-equilibrated for 7 min.

The MS setting was identical to Chen et al. (2022): capillary 2.5 kV, source temperature 120°C, sampling cone 45, source offset 80, desolvation gas flow 800 L/h at 350°C, cone gas flow 50 L/h, nebulizer gas flow 6.5 bar. The TOF analyzer was operated at Resolution mode and the data acquisition mode was FAST-DDA. The scan time was 0.2 s and the mass range for MS^1^ and MS^2^ was *m*/*z* 100–2,000 and *m*/*z* 50–2,000, respectively. Ions with the top 5 intensity and >20,000 total ion chromatogram counts were fragmented by collision-induced dissolution (CID) to obtain product ion spectra. For lower-intensity ions, a real-time dynamic exclusion was enabled. Ramped collision energy from 10 V to 55 V for low mass and 15 V to 65 V for high mass was used to obtain the MS^2^ spectra. The MS was calibrated before the analysis using sodium iodide solution (*m*/*z* 50–2,000; residual mass error < 0.5 ppm). A leucine enkephalin solution (*m*/*z* = 556.2771 for [M + H]^+^) was used for real-time calibration (scan time 0.2 s, 20 s interval).

The continuum raw data was centroid using MassLynx v 4.1 software and then transformed into mzML format using Proteowizard v 3.0.20247. The mzML files were then imported into MS-DIAL v4.9 software for further lipidomic analysis (Tsugawa et al., 2020). The retention time (RT) drifts between the cohort samples were correlated using internal standards with the RT deviations <0.8 min. Peak detection and integration were performed in MS-DIAL v4.9 with a minimum peak height of 3,000 and mass slice width of 0.1 Da. The detected peaks were aligned against a QC sample as reference with RT tolerance 0.7 min and MS^1^ tolerance 0.05 Da. The mass features detected in samples lower than 10 folds of that present in blanks were filtered.

An in-house *in silico* archaeal lipid MS^2^ library was used for feature annotation and lipid identification in MS-DIAL v4.9. The *in silico* library was made based on and modified from the LipidBlast Templates (Kind et al., 2014), with fragmentation rules obtained from the published MS^2^ data of archaeal lipids (Sprott, 1992; Yoshinaga et al., 2012; Elling et al., 2016; Bale et al., 2019; Kropp et al., 2022) and the data obtained under our equipment setting. Lipids were annotated with mass error within 0.01 Da for MS^1^ and 0.05 Da for MS^2^ and total scores over 70% matching to the library were considered as identified compounds. The detailed MS^2^ of representative compounds were shown in Supplementary Figures S1–S3. The annotation results were further manually confirmed, and our results indicated that the total score of 70% as threshold was an optimized balance of the annotation coverage and accuracy.

The [M + H]^+^ and [M + NH_4_]^+^ ions were included in the peak integration. Lipid abundance was semi-quantified by the internal standard 1,2-dinonadecanoyl-sn-glycerol-3-phosphocholine (Kellermann et al., 2016), while the response factors of different compounds were not determined due to the lack of commercial standards for most compounds. Data of logarithmic relative abundance of lipids were scaled by samples and then used for cluster analysis by pheatmap package v 1.0.12 (Kolde, 2012) in *R* v 4.21 (Team, 2013). Complete linkage method was used for cluster analysis, while single linkage, Ward. D linkage and average linkage methods were also tested to confirm the consistency of the cluster results (Supplementary Figure S7). The principal component analysis (PCA) and partial least squares discrimination analysis (PLS-DA) analysis were performed in MetaboAnalyst v5.0.^1^ The data were normalized by sum and log transformed during the submission, and the default setting was applied for each analysis (Pang et al., 2022).

### 2.4 Feature-based molecular network construction

MS^2^ spectral summary and feature quantification were exported from MS-DIAL v4.9 and then imported for the Feature-Based Molecular Networking analysis on Global Natural Products Social Molecular Networking (GNPS) following the detailed documentation (Wang et al., 2016; Nothias et al., 2020; Ding et al., 2021). Mass tolerance was set to 0.05 Da for precursor and MS^2^ fragment ions. The MS^2^ spectra were filtered by collecting the top 6 fragment ions within a 50 Da window and discarding fragments within 17 Da of the precursor. Spectrum pairs with a cosine score above 0.7 and sharing 6 peaks were considered linked pairs. Spectra were further annotated in the GNPS library with the same filter (Horai et al., 2010; Wang et al., 2016; Mohimani et al., 2018). Network visualization was performed in Cytoscape v 3.9.1 software (Shannon et al., 2003). Nodes involved in blank samples or with *m*/*z* < 500 (to collect features that are more likely to be lipids), and networks containing less than 4 nodes were filtered. After the filtration, only one annotated features of G-1,2-Didecanoyl PC (*m*/*z* = 566.381) was remained. SIRIUS v 5.6.2 was used for assistance in compound formula indication (Dührkop et al., 2019).

### 2.5 Phylogenetic analysis

An aliquot of the collected biomass was used for DNA analysis. DNA were extracted using Fast DNA SPIN KIT (MP Biomedicals, OH, United States) following the manufacturer’s protocol. The extracted DNA were sent to Sangon Biotech for Sanger sequencing. Primer pair of Arch 21F: TTCCGGTTGATCCYGCCGGA and 1492R: TACGGYTACCTTGTTACGACTT was used to acquire full-length 16S rRNA gene sequences (Oyama et al., 2018). SeqMan v7.1.0 was used for sequence assembly (Swindell and Plasterer, 1997). Ten extra 16S rRNA gene sequences were obtained from NCBI as reference sequences (Supplementary Table S1). All sequences were imported into MEGA 11 for further analysis (Tamura et al., 2021). The sequences were aligned using the built-in MUSCLE algorithm and the gaps were manually removed (Edgar, 2004). A neighborhood joining phylogenetic tree was constructed with 1,000 bootstrap tests (Saitou and Nei, 1987).

## 3 Results

### 3.1 Lipid identification of Halobacteria

**Figure 2:**
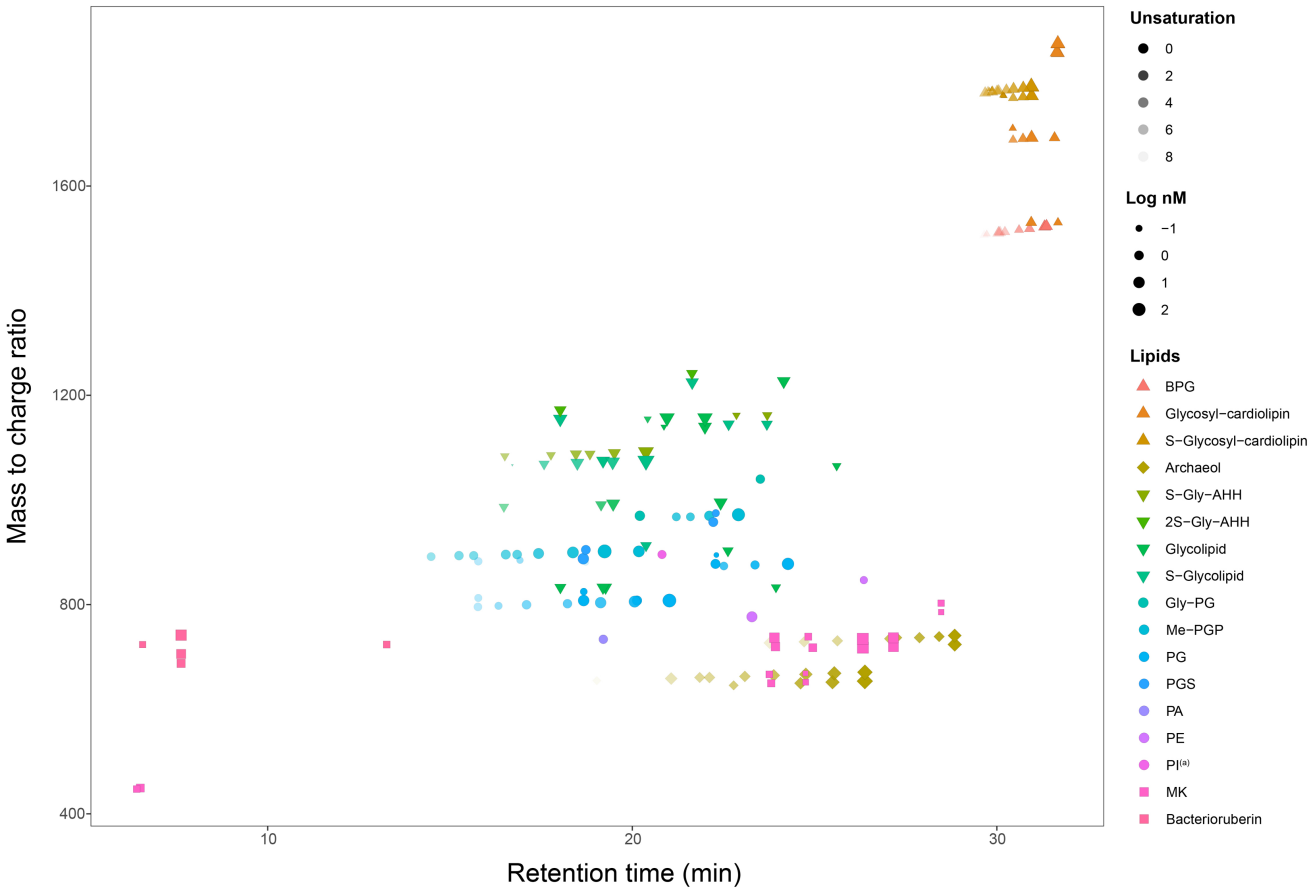
A map of all 162 lipids features (107 unique lipids in 17 classes, including AR, IPLs, cardiolipin, quinone, and bacterioruberin) from seven cultured Halobacteria. (a) Tentative identification as the MS^2^ spectrum lack some of the characteristic fragments.

A total of 192 peaks were annotated against an in-house *in silico* archaeal lipid database after peak alignment using MS-DIAL v4.9. The annotated peaks were manually curated to reduce false positive annotations and interference peaks from in-source fragmentation, finally resulting in 162 correctly annotated features. Regardless of different isomer and adduct forms, we finally had 107 unique lipids (Figure 2, Table 2). These lipids included a large range of lipid diversity, composing of 16 core structure of diethers, 30 phospholipids (2 phosphatidyl glycerolhexose, Gly-PG; 9 Me-PGP; 13 PG; 2 phosphatidyl glycerolsulphate, PGS; 1 phosphatidyl acid, PA; 2 phosphatidyl ethanolamine, PE; 1 tentative phosphatidyl inositol, PI^(a)^), 27 glycolipids (8 sulphated/disulphated glycosyl aminohexanehexaol, S-Gly-AHH / 2S-Gly-AHH; 10 non-sulphated glycolipids; 9 sulphated-glycolipids), 22 archaeal cardiolipins (7 bisphosphateglycerol, BPG; 5 non-sulphated glycocardiolipins; 10 sulphated-glycocardiolipins), 8 quinones of MK, and 4 bacterioruberin (Figure 2). Representative MS^2^ spectra and the matches with reference spectra of the *in silico* archaeal lipid library are provided in the Supplemental material (Supplementary Figures S1–S3, Supplementary Table S2). One feature (*m*/*z* = 895.711) was tentatively identified as PI, which showed characteristic ions of phospholipids but lacked a fragment ion resembling the polar head group (*m*/*z* = 261.038).

**Table 2:** Lipid profiles of seven Halobacteria strains.

Order	*Halobacteriales*	*Haloferacales*			*Natrialbales*		
Genus/species	*Haloarcula argentinensis*	*Haloferax mediterranei*	*Haloferax larsenii*	*Haloferax volcanii*	*Haloterrigena turkmenica*	*Natrialba asiatica*	*Natrinema gari*
Unsaturation		>10%			1 ~ 10%		
C_25_ chain					>10%		
MK	***	***	***	***	***	***	***
Archaeol	***	***	***	***	***	***	***
Me-PGP	***	***	***	***	***	***	***
PG	***	***	***	***	***	***	***
Bacterioruberin	***	*	***	***	***		***
PGS	***	***					***
TGD	***						***
BPG	***	*	***	*			
TGD-PA	***						
DGD-PA	***	***	***	***			
S-DGD-PA		***	***	***			*
S-DGD		***	***	***	***	***	***
DGD	*	***	***	***	***	*	***
S-Gly-AHH		***	***	***	***	***	*
PE		***	***	*	***	***	***
MGD			*		***	***	***
PI^a^		***	*	*			
2S-DGD					***	***	
2S-Gly-AHH					***	***	
Gly-PG				*	*		***
PA	*	*	*	*	*	*	*
MGD-PA	*	*	*	***			
S-MGD		*	*	*	*		

***Relative abundance > 1/1,000; *relative abundance > 1/10,000. Identification of content less than 1/10,000 is suspectable due to the background noises.

a Tentative identification as the MS^2^spectrum lacks some of the characteristic fragments.

### 3.2 Comparative analysis of Halobacteria lipidome

#### 3.2.1 Composition of diether core lipids

For a complete investigation of the core membrane lipid composition of Halobacteria, the detected core lipids were manually combined with the IPLs that share the same core lipids. The core lipid (including archaeol that are identified directly or involved in the IPLs) of the seven Halobacteria strains primarily consisted of diether core structures with two fully saturated C_20_ chains (AR_C_20_:C_20_, short for AR, 47.71–99.62%), while diether variant structures of unsaturated and extended chain length (C_25_) were also identified in some strains as expected. The degree of unsaturation in archaeol ranged from 0 to 8, and the extended archaeol were composed of a C_20_ chain and a C_25_ chain (AR_C_20_:C_25_, short for Ext-AR), whereas di-extended archaeol of two C_25_ chains (AR_C_25_:C_25_, diExt-AR) was not detected in any halobacterial strains in this study.

**Figure 3:**
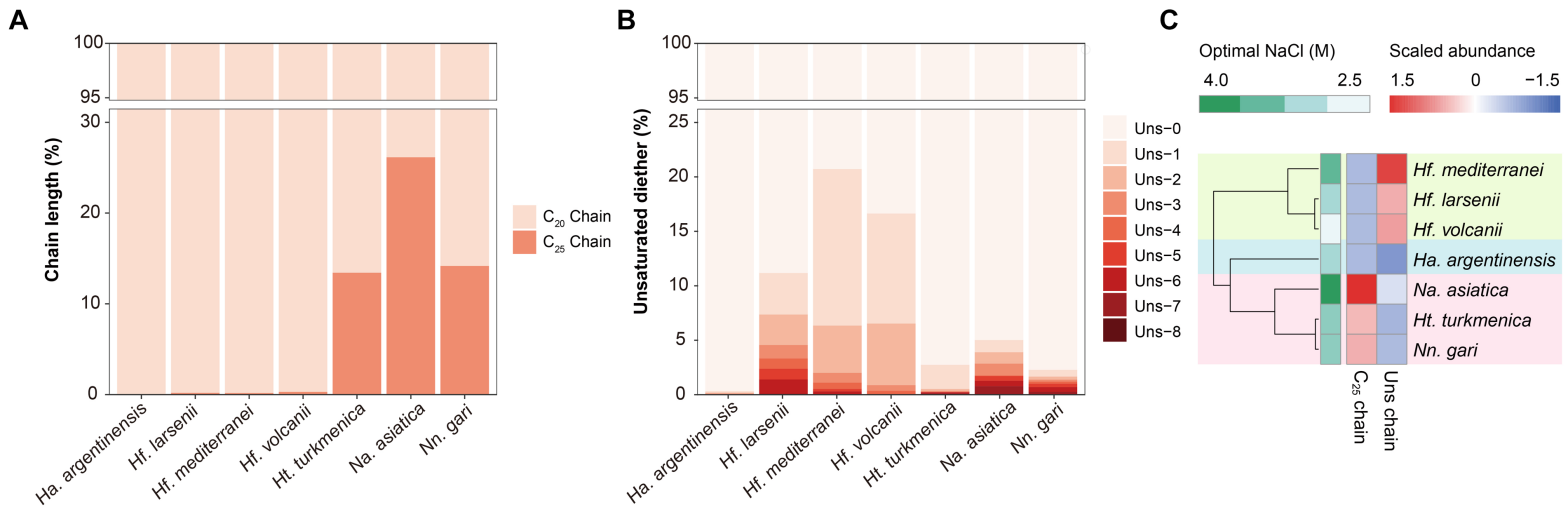
Different variables of core lipids, expressed in percent, across the seven Halobacteria cultures, including **(A)** unsaturation in diether molecules and **(B)** isoprenoid chain length. **(C)** Complete linkage cluster analysis of the variation in core lipids. Proportion of unsaturated lipids in total lipids and proportion of C_25_ chains in total chains are used in this analysis.

The distribution of unsaturated diether lipids varied among the three orders of Halobacteria (Figure 3B). Particularly, *Haloferacales*, which comprises three strains belonging to the genus *Haloferax*, exhibited the highest fraction of unsaturated diether lipids (11.17–20.72%) compared to *Natrialbales* (2.27–5.01%) and *Ha. argentinensis* (the sole strain in the order *Halobacteriales*, 0.32%). *Hf. mediterranei* produced the most abundant unsaturated lipids (20.72%) in *Haloferax* with predominantly low degrees of unsaturation (unsaturation <4, 97.44%). Additionally, the unsaturated diether lipids synthesized by *Hf. volcanii* were primarily low-unsaturated (99.61%), while *Hf. larsenii* exhibited significantly higher production of polyunsaturated lipids compared to its counterparts (unsaturation ≥4, 21.27%). Among the *Natrialbales* order, *Na. asiatica* and *Nn. gari* were characterized by the highest production of polyunsaturated lipids (unsaturation ≥4, 33.93 and 44.43% respectively). Although less capable of highly unsaturated lipid production among *Natrialbales*, *Ht. turkmenica* still yielded more polyunsaturated lipids (9.05%) than *Hf. mediterranei* and *Hf. volcanii*.

The Ext-AR was detected abundantly in the *Natrialbales*, accounting for 13.44–26.16% of total archaeol chains. This corresponded to an Ext-AR content in the range of 26.88–52.33% of the total core lipids (Figure 3A). While traces of Ext-AR were also detected in other strains, its abundance was less than 0.3%. These results are consistent with previous studies emphasizing the predominant occurrence of Ext-AR in *Natrialbales* (Vandier et al., 2021). Remarkably, under the same culture conditions, the core lipid compositions exhibited significant variations among the seven strains. Cluster analysis of these lipids successfully differentiated the strains at the order level, indicating a strong connection between phylogeny and core lipid composition (Figure 3C). Notably, the strains with the highest optimal salt demand, *Hf. mediterranei* and *Na. asiatica,* produced the largest amounts of unsaturated lipids or Ext-AR, leading to independent branches of both strains in their respective groups (Figure 3C).

#### 3.2.2 Composition of intact polar lipids and non-bilayer forming lipids

**Figure 4:**
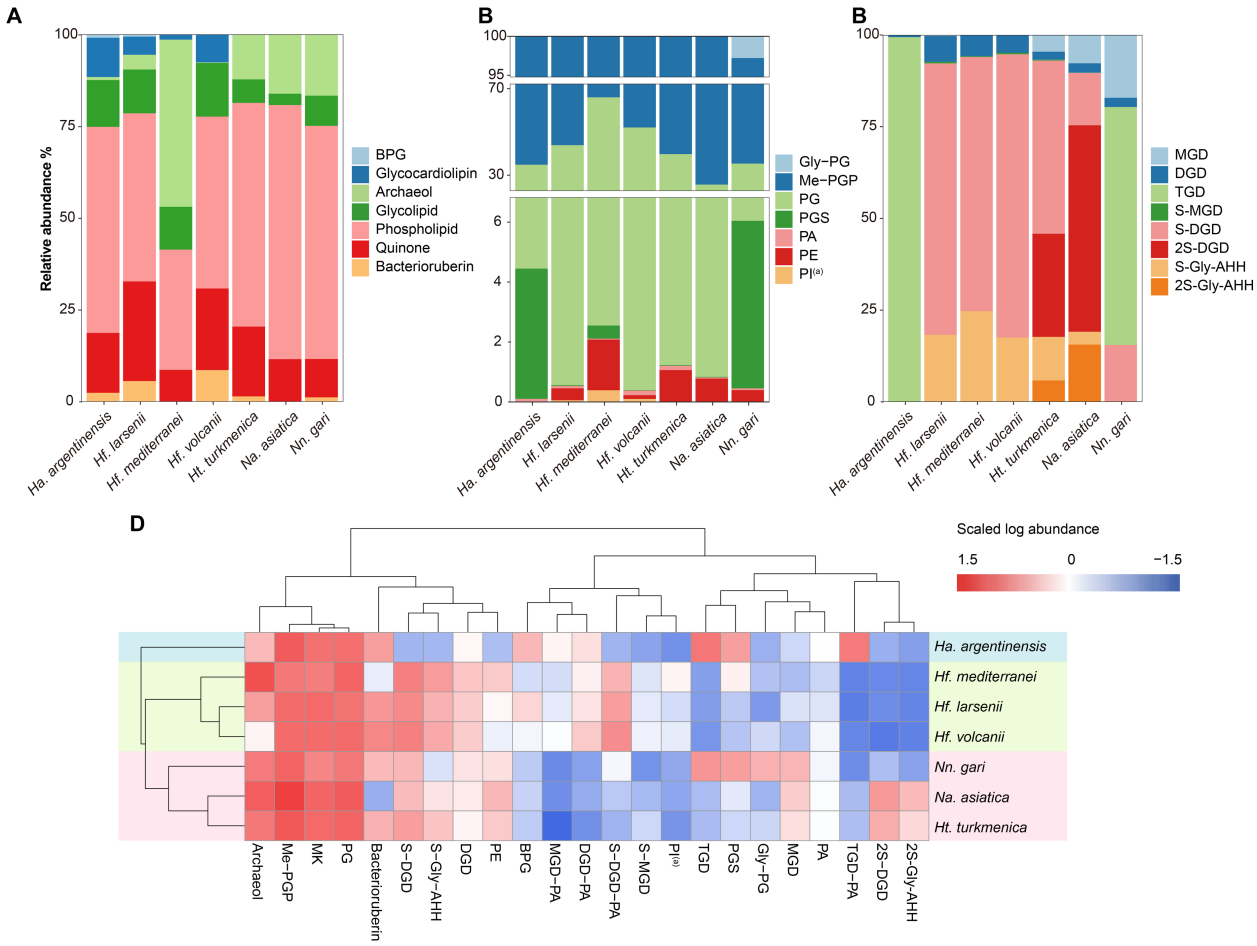
Lipidome composition of seven cultured Halobacteria, including **(A)** composition of total lipids, **(B)** composition of glycolipids (including lipids with a reported structure of S-C_12_H_23_NO_10_ (Bale et al., 2019), which is named as sulphated Gly-AHH in this study) and **(C)** composition of phospholipids. **(D)** Complete cluster analysis of the lipidome using grouped lipids as an element. The lipid classes examined include BPG: bisphosphatidylglycerol; MGD: monoglycosyl diether; DGD: diglycosyl diether; TGD: triglycosyl diether; S-MGD: sulfo-monoglycosyl diether; S-DGD: sulfo-diglycosyl diether; 2S-DGD: disulfo-diglycosyl diether; Gly-PG: phosphatidylglycerolhexose; Me-PGP: methylated phosphatidylglycerolphosphate; PG: phosphatidylglycerol; PGS: phosphatidylglycerolsulfate; PA: phosphatidic acid; PE: phosphatidylethanolamine; PI: phosphatidyl inositol; MK: menaquinone. (a): Tentative identification, the MS^2^ lack some of the characteristic fragments.

The intact polar lipids (phospholipids, cardiolipins and glycolipids) and non-bilayer forming lipids (quinone and bacterioruberin) were found in the strains but with varying fractions. Phospholipids comprised the largest fraction of the total lipids in most strains (45.70–69.17%, Figure 4A), except for *Hf. mediterranei* (32.76%). Among all strains, Me-PGP and PG were the predominant phospholipids (34.03–74.35% and 24.81–63.41%, respectively), which aligned with the observation of previous studies (Kellermann et al., 2016). PA, a biosynthetic intermediate for other phospholipids (Corcelli, 2009), was detected in all strains but only present in minor fractions (0.13–0.91%). The distribution of other phospholipids, such as PE, PI^(a)^, and PGS, showed taxonomic specificity. PE was found exclusively in strains of *Haloferax* and *Natrialbales* (0.06–0.65%), while PI^(a)^ was specific to *Haloferax* strains (0.03–0.12%). PGS was detected in *Ha. argentinensis*, *Hf. mediterranei* and *Nn. gari* with contents of 2.44, 0.14 and 3.54%, respectively (Figure 4B, Table 2).

The production of cardiolipins was higher in three *Haloferax* species and *Ha. argentinensis.* In *Natrialbales*, only a small amount of glycocardiolipins (sulfate diglycosyl diether-phosphatidyl acid, S-DGD-PA) was detected (0.44‰) in *Nn. gari*. Comparing to *Haloferax* (1.42% in *Hf. mediterranei*, 5.59% in *Hf. larsenii* and 7.61% in *Hf. volcanii*), *Ha. argentinensis* exhibited a significantly higher cardiolipin content (11.60%). In all cases of stains producing cardiolipin, glycocardiolipins were predominant over BPGs (1.41–10.67% comparing to 0.02–0.61%, Figure 4A).

Glycolipids were found to be less abundant than phospholipids and made up 2.50–12.74% of the total lipids. The compositions of glycolipids also exhibited taxonomic specificity in these seven strains. The three *Haloferax* strains displayed similar glycolipid profiles, with sulfate diglycosyl diether (S-DGD) being the dominant glycolipid (69.30–77.18%), followed by S-Gly-AHH (17.53–24.70%), diglycosyl diether (DGD) (4.80–7.03%), and minor sulfate monoglycosyl diether (S-MGD) (0.21–0.43%, Figure 4C, Table 2). *Ht. turkmenica* and *Na. asiatica* also produced diglycosyl-based glycolipids as major components but were also capable of producing bis-sulfate glycosyl moiety, forming disulfate diglycosyl diether (2S-DGD) (28.10 and 56.29%) or 2S-Gly-AHH (5.82 and 15.59%). *Na. asiatica* showed a higher abundance of bis-sulfate glycosyl lipids than *Ht. turkmenica*. Both strains produced more monoglycosyl diether (MGD) compared to *Haloferax*. However, *Nn. gari*, despite belonging to the same order, displayed distinct glycolipids. It lacked the bis-sulfate glycosyl moiety, and S-Gly-AHH was only present in small amounts. Instead, triglycosyl diether (TGD, 64.83%) was the primary glycolipid in *Nn. gari*, along with MGD (17.12%), S-DGD (15.34%), and DGD (2.53%). In *Ha. argentinensis*, TGD was the predominant glycolipid, accounting for almost all the glycolipids detected (99.36%) (Figure 4C).

Respiratory quinones were also identified in all halobacterial strains. The dominating compounds were polyunsaturated MKs with 8 isoprenoid units (MK_8:8_-MK_8:7_). The MKs were abundant in all cultures with a maximal abundance being detected in *Hf. larsenii* (27.08%) and lowest in *Hf. mediterranei* (8.62%). Notably, bacterioruberin, a red carotenoid pigment, is a well-known biomarker for Halobacteria (Oren, 2002); however, our results showed this compound was not universally produced in Halobacteria. Five strains (except for *Hf. mediterranei* and *Na. asiatica*) showed high abundance of bacterioruberin, ranging from 1.20 to 8.62%. In contrast, *Hf. mediterranei* exhibited only a minor amount of bacterioruberin at 0.04% (Figure 4A, Table 2). Notably, bacterioruberin was entirely absent in the culture of *Na. asiatica*, which has been reported as a pigment-free species (Hezayen et al., 2001). Thereby, the detection of bacterioruberin was in line with the observation of a pink or red color exhibited in the cultures of the other five strains.

The PCA analysis using the grouped identified lipids (Supplementary Figure S8A) revealed that most strains could be differentiated based on the variation in lipid groups, except *Hf. larsenii* and *Hf. volcanii*, which exhibited similar patterns as expected (Figure 4). The three *Natrialbales* strains showed great divergence in the PCA plot compared to *Haloferax* strains. To investigate the relationship between lipid groups and DNA-based phylogeny, we further performed cluster analysis using lipid groups as elements. The results demonstrated a clear separation of three groups at the order level, with three *Haloferacales*, three *Natrialbales*, and the single *Halobacteriales* forming distinct clusters (Figure 4D). Compared to the cluster of core lipid variation alone (Figure 3C), there was a shift in the position of the three groups. In the grouped identified lipid cluster analysis, *Ha. argentinensis* appeared as a distinct branch (Figure 4D), whereas it was more closely related to *Natrialbales* in the core lipid cluster analysis (Figure 3C). In *Natrialbales*, *Nn. gari* formed a separated branch, replacing *Na. asiatica*. Obviously, both the phospholipid and glycolipid compositions supported a distinct lipidome of *Nn. gari* among *Natrialbales*. However, the distant relationship of *Nn. gari* contradicted the phylogenetic relation, which suggested an independent branch for *Na. asiatica* (Figures 1, 4D).

### 3.3 Extended identification of lipids using feature-based molecular network analysis

#### 3.3.1 Lipids involved in the networks

**Figure 5:**
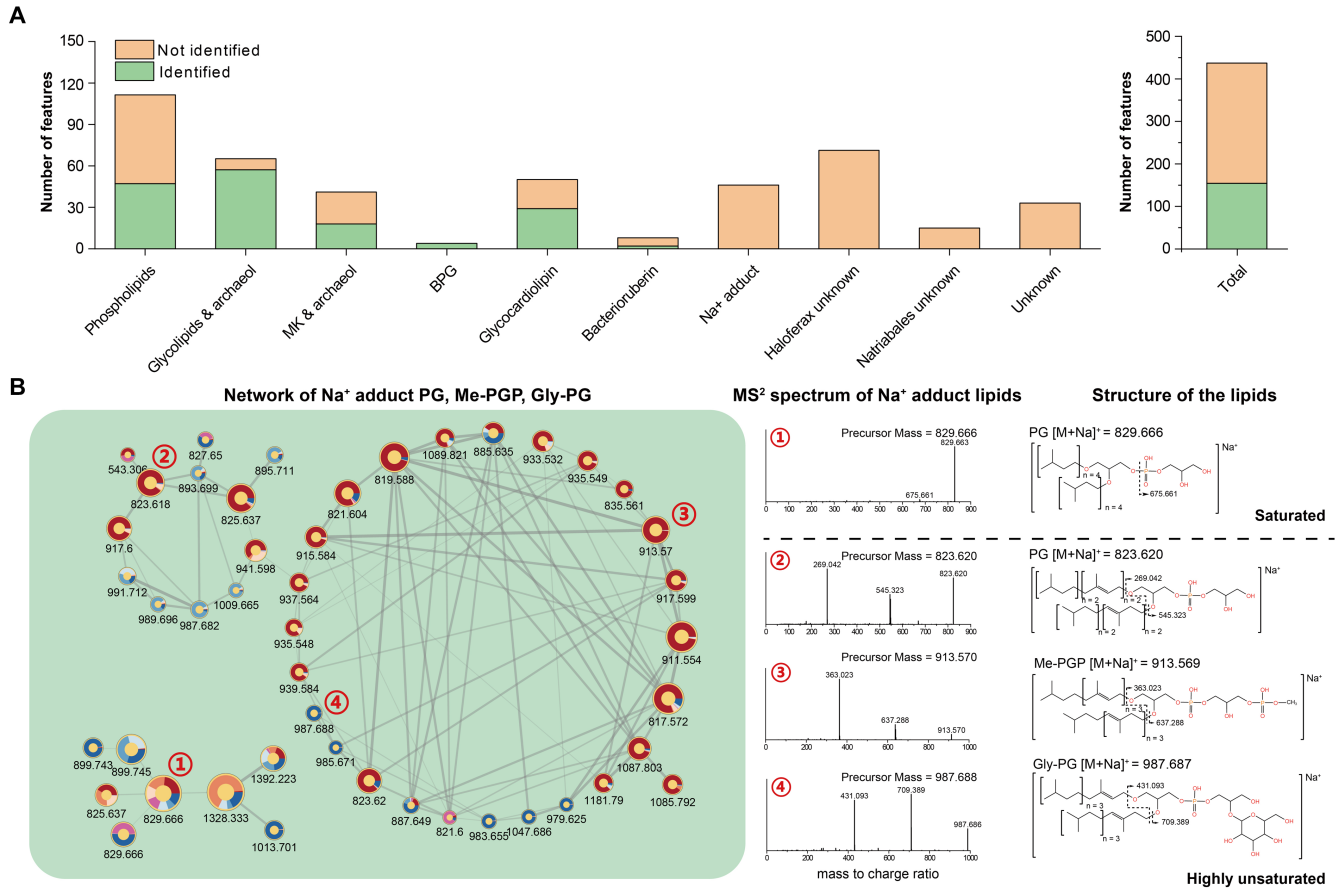
**(A)** Quantification of lipid features within each network; **(B)** Network of PG, Me-PGP, and Gly-PG with Na^+^ adduct.

The feature-based molecular network comprised 444 lipid features after construction and filtration. Of these, 157 had been described previously and were thus identified using our in-house library (Figure 5A). The remaining 287 unknown features were either analogs of the identified ones (122) or new structures (165) that formed different networks on their own (Supplementary Figure S4). All networks were then manually assigned into 10 categories based on the major constituent of known lipids and occurrence in different strains.

#### 3.3.2 Resolution of features within networks

By analyzing features within the network, the phospholipids-related molecular networks involved 111 lipid features, and 64 of them remain unidentified. The molecular networking and further analysis of their MS^2^ spectra revealed that most of these features were closely related to the identified phospholipids and contained the characteristic fragments of the known phospholipids. These features might result from new adduct forms of PG, Me-PGP, and Gly-PG in the analyzer, or in-source fragments. We tentatively identified these features as phospholipids that have lost one isoprenoid chain, highly unsaturated lipids attracting an extra chain or even another lipid, and novel adduct forms with short-chain alkylamine (Supplementary Figure S5).

We further identified networks consisting of unknown features (Figure 5B, Supplementary Figure S4). These features can be assigned as compounds of PG, PGP-Me, and Gly-PG with sodium adduct ions ([M + Na]^+^) based on manual examination of MS^1^ and RT. Previous studies showed that archaeal lipids with sodium adduct ions ([M + Na]^+^) tended to resist fragmentation under CID, making it difficult to provide structural information. Therefore, sodium adducts were not included in our in-house *in silico* library for their poor resolvability, which may lead to false annotation. However, we observed that the MS^2^ spectral of unsaturated archaeal diethers with sodium adduct ions ([M + Na]^+^) could produce fragments ions associated with the intact unsaturated chain loss (Figure 5B), which may provide additional structural information for these lipids.

Finally, some sub-networks were entirely composed of unknown lipids. They presented a strong taxa specificity and could be defined as *Haloferax*-dominant unknown lipids or *Natrialbales*-dominant unknown lipids. This indicated that Halobacteria of *Haloferax* and *Natrialbales* were likely to biosynthesize unique lipids with highly chemotaxonomic potential, which should give traces for halobacterial chemotaxonomy; however, their structures still require elucidation and identification (Supplementary Figure S6).

#### 3.3.3 Enhancing the lipidomic capability of chemotaxonomy by network-coordinated features

**Figure 6:**
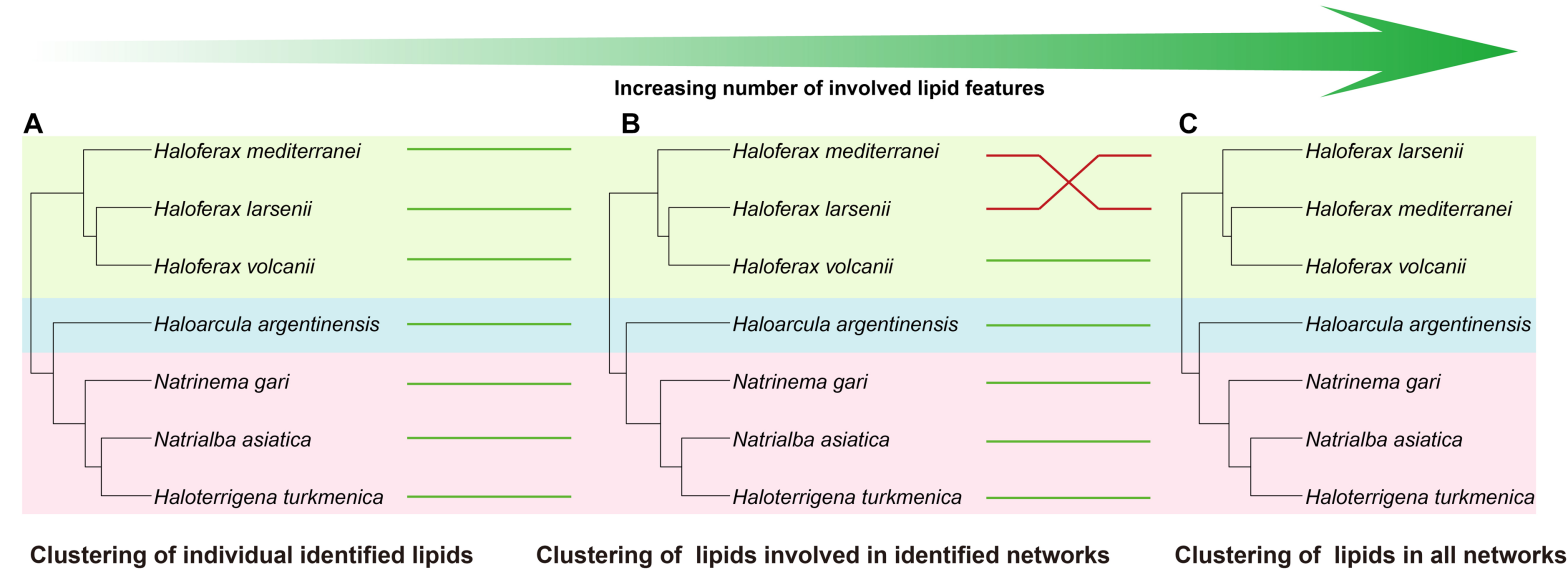
Cluster analysis using different levels of features, including **(A)** all identified lipids, **(B)** lipids involved in identified networks, and **(C)** lipids in all networks. Four cluster methods (complete linkage; single linkage; Ward. D linkage; average linkage) are used for each analysis to ensure consistency (Supplementary Figure S7). Strains in different clusters are linked using green or red lines. Green line refers to consistent cluster result while the red line refers to inconsistent cluster result.

Comparing to the PCA analysis using lipid groups, the analysis using network-involved features resulted in a better separation between and within the three groups (Supplementary Figures S8B,C). The PLS-DA analysis revealed that several features involved in network showed significant capability in distinguishing the three halobacterial orders. Among these features, two features in unknown network showed the highest variable importance in the projection (VIP) scores, and 7 unidentified features, two of which were located in the *Natrialbales*-dominant unknown sub-network, ranked among the top 15 VIP scores (Supplementary Figure S9H). This highlighted the importance of incorporating new features to uncover the chemical distinction between strains. To further investigate the chemotaxonomic potential of the lipidomic approach, we performed cluster analysis using individual lipid features (Figure 6A). Compared to the lipid group cluster analysis, the individual identified lipids cluster showed minimal differences except for the positioning of *Ha. argentinensis* (Figures 4D, 6A), which was consistent with core lipids cluster (Figure 3C). The individual lipid cluster provided additional information regarding the core lipids that was not apparent in the grouped cluster analysis, revealing a less diverse lipid profile in *Ha. argentinensis* and contributing to its distinct chemotaxonomic status.

In previous cluster analyses, *Hf. mediterranei* appeared as a distant relative within the *Haloferax* group, possibly due to its high content of archaeol in total lipids and the presence of large amounts of unidentified lipids. When lipid features involved in the identified networks were used, which encompassed all possible analogs of known lipids, there was no significant variation observed in the cluster topology (Figure 6B). However, when all features from the networks including those in unknown networks were introduced, changes occurred in the relationship between *Haloferax* strains. *Hf. larsenii* emerged as the distant relative, which better aligned with the phylogenetic relation (Figures 1, 6C).

## 4 Discussion

### 4.1 Lipidome profiling strengthened by high-resolution MS and informatic data processing

Considerable efforts have been devoted to reveal archaeal lipid diversity with the developments of analytical approaches. For example, Elling et al. (2017) successfully identified 118 structurally different lipids in 10 thaumarchaeal cultures, which provided an elaborate database of their membrane lipid composition, enabling us to compare the distribution of lipids in different lineages. Bale et al. (2019) identified a wide range of known and novel lipid compounds in 13 strains of halo(alkali)philic methanogens and Halobacteria, which revealed structural diversity and chemotaxonomic potential of archaeal lipids. However, the traditional manual identification of archaeal lipids analyzed by HRMS is still challenging given the demand for processing and identifying large amounts of lipid features, which may substantially affect the efficiency and accuracy of the analysis.

Combining the untargeted lipidomic approaches and the *in silico* mass spectral library, we were able to simultaneously determine a wide range of structurally different lipids. After peak alignment, a total of 6,470 mass features were detected, with 2,347 features carrying MS^2^ spectra, of which 162 features were annotated with our *in silico* library, resulting in an annotation coverage of 6.90%. The annotation results referred to 107 unique lipids, encompassing the majority of lipids previously reported in Halobacteria. These included unsaturated and extended diether core structures; IPLs of PG, Me-PGP, DGD and S-DGD; cardiolipins of both BPG and glycocardiolipins; non-bilayer forming lipids of MK and bacterioruberin (Kamekura and Kates, 1999; Ventosa et al., 1999; Hezayen et al., 2001; Sprott et al., 2003; Xu et al., 2007; Tapingkae et al., 2008; Minegishi and Kamekura, 2009; Ventosa et al., 2009; Elling et al., 2016).

The application of high-resolution mass spectrometry and bioinformatical approaches has allowed us to identify lipids that may be a minor component but are still important in archaea. For example, we were able to detect diether IPLs with PE and PA headgroups in halobacterial strains. PA was a universal intermediate compound in the biosynthesis of phospholipid but has often been overlooked in earlier research (Mullakhanbhai and Larsen, 1975; Ihara et al., 1997). Additionally, PE has been suggested to be absent in Halobacteria (Tenchov et al., 2006), possibly due to the low sensitivity of instrumentation used in previous studies. Recent studies, however, have detected PE in several strains of Halobacteria facilitated by HRMS (Kellermann et al., 2016; Sorokin et al., 2019). These findings have expanded our knowledge of the halobacterial lipidome and highlight the power of bioinformatic tools supervised by manual corrections in lipidome analysis.

### 4.2 The prospect of feature-based molecular network analysis

Feature-based molecular network techniques are an efficient tool in elucidating lipidomics data by linking features with similar MS^2^ spectra. This approach enables the visualization and manually annotation of molecular networks, presenting the distribution of features in grouped samples, *m*/*z* value, and structural similarity between different lipid feature or lipid groups (Ding et al., 2021). Our results suggest that FBMN analysis also allows for the characterization of specific unknown features that are potentially crucial in some Halobacteria. Although the detailed structure of those features cannot be directly determined, their structural characteristics can be inferred to some extent according to the relationships in network or using different analytical methods. For example, despite not being identified previously, the feature with an *m*/*z* value of 852.745, involved in the network of phospholipids, is likely a novel phospholipid compound (Supplementary Figure S5A). Additionally, some unassigned lipid groups can form identical networks with their known analogs, shedding light on the identification of new lipid types.

In this study, the resulting network comprises 444 features and accounts for 18.92% of the total MS^2^ features, far more than the identified features (162 features, 6.90%). This means that lipidome data can be better elucidated by introducing network analysis. On the basis of network analysis, a fragmentation rule for unsaturated IPLs with Na^+^ adduct could be concluded, i.e., the sequential losses of two intact unsaturated isoprenoid chains (Figure 5B). Additionally, the phospholipid network revealed the features of short-chain alkylamine adducts and unsaturated phospholipids that exhibit a propensity to attract extra unsaturated chains or lipids (Supplementary Figure S5). Although these features do not correspond to new lipids, it provides new perspectives to track known lipids in different adduct forms.

In addition, several unannotated networks were found, which contained features with MS^2^ characteristics indicating the loss of one or two isoprenoid chains. This demonstrated the presence of novel IPLs or other lipids containing isoprenoid chains. Moreover, based on their distributions among different species, some of these unknown features are also taxa-specific and potentially contribute to chemotaxonomy (Supplementary Figures S4, S6). Despite the structural analysis of these features are not available, formula predictions can be performed using the MS^2^ information in SIRIUS v5.6.2 (Supplementary Figure S6), which allowed for a primary prediction of element composition of the new lipid compounds. By introducing network features into the cluster analysis, especially those unknown components, a change in the cluster topology occurred, making the chemotaxonomy to better fit the phylogeny inferred from the 16S rRNA genes (Figures 1, 6). This highlights the improved effectiveness of lipid chemotaxonomy when incorporating unknown lipid features. Overall, the application of molecular networks has the potential to drive a new paradigm in lipidomic research, opening new avenues for exploration on archaea (see below).

### 4.3 Lipidomic potential for phylogenetic classification of Halobacteria

Attempts for chemotaxonomy analysis of microorganisms have been made for a long period (Fenselau, 1993; Vandamme and Sutcliffe, 2021). A recent Raman spectroscopy application on cell classification reveals distinct chemotaxonomic pattern among halophilic archaea, thermophilic archaea, Thaumarchaeota and bacteria (Wang et al., 2021). Lipids are one of the most important chemotaxonomic biomarkers and have been widely used in identifying new isolated bacteria and archaea strains (Elling et al., 2017; Villanueva and Coolen, 2022; Edwards, 2023). In previous studies, several taxa-specific lipids of archaea have been identified and lipid component-based phylogenetic inference was used to indicate the presence of certain taxa. For example: PGS head group was suggested to be biomarkers for neutrophilic haloarchaea and *Methanothermaceae* was unique for having inositol as the sole phospholipids head group (Koga et al., 1993; Bale et al., 2019).

In this study, we illustrated that seven halobacterial strains, including three strains from *Haloferax* genus, could be chemotaxonomically differentiated through a comprehensive analysis of their lipidomes (Supplementary Figure S8). Archaeol, PG, Me-PGP and MK are common lipid components identified in all 7 cultures of Halobacteria, while other lipid constitutions, such as diverse polar head groups and core lipid structures, exhibit selective distribution among the phylogenetic taxonomy (Table 2). Both lipid modification of unsaturation and extended length on carbon chains in diether core structures are suggested to be adaptive strategies under hyper-salinity stress. Our results revealed that different halobacterial groups, may choose distinct membrane adaptation strategy to salinity stress. For instance, *Haloferax* chooses the strategy by regulating the unsaturation of carbon chains, while *Natrialbales* by extending the length of carbon chains, which can be used to distinguish the three Halobacteria groups. The phospholipid composition in Halobacteria is relatively consistent, but a few minor components such as PE and PI are present in specific groups. Whereas the glycolipids are quite diverse, varying in the number of glycosyl moiety and sulfate moiety among different strains. Bacterioruberin is absent in *Na. asiatica* and has a low amount in *Hf. mediterranei*, which are extreme salt-adapted strains (Merino et al., 2019) that have the highest optimum NaCl concentrations (3.4 M and 4.0 M, respectively) among the 7 strains (Figure 3C) (Hezayen et al., 2001; Ventosa et al., 2009). Accordingly, we inferred that strains thriving under such extreme salt conditions may not favor the production of bacterioruberin.

Among the seven studied strains, *Ha. argentinensis* has the simplest lipidomic diversity, with only a few lipids containing unsaturation or extended chain detected in its core lipids. Interestingly, two novel phospholipids (PE and PI) that were identified in other strains were absent in *Ha. argentinensis*. Instead, we observed that the dominance of triglycosyl diether-phosphatidylglycerol (TGD) is a unique feature of *Ha. argentinensis*. TGD is also detected in *Nn. gari*, but the incapability of *Nn. gari* to produce cardiolipin makes triglycosyl diether-phosphatidyl acid (TGD-PA) a characteristic of *Ha. argentinensis*. Another report of TGD-related cardiolipin is sulfate triglycosyl diether-phosphatidyl acid (S-TGD-PA) in *Halobacterium salinarum* (Corcelli et al., 2000), which belongs to the same order as *Ha. Argentinensis*, but a different family from *Ha. Argentinensis* (Figure 1). Based on these findings, TGD-PA was likely to be a specific biomarker for order *Halobacteriales*.

The lipidome of *Haloferax* genus indicated their ability to produce the unsaturated core lipids, with PI headgroup serving as a featured intact polar lipid. All three strains within this genus also have the ability to produce minor PE, while PGS were only present in *Hf. mediterranei*. The composition of glycolipids in the three strains was similar, with S-DGD being the major component, followed by S-Gly-AHH and DGD. The main cardiolipin lipids in the three *Haloferax* strains were glycocardiolipins, which was similar to *Ha. argentinensis* and strains in *Haloferax* were capable of synthesizing glycocardiolipins in sulphated form.

The three *Natrialbales* strains shared the ability to produce abundant lipids with C_25_ chain, as well as moderate amounts of unsaturated lipids, PE, and sulfated glycosyl moiety. However, they lacked the ability to produce PI^(a)^. The main difference among these stains were the biosynthesis of diglycosyl-based glycolipids as their main glycolipids in *Ht. turkmenica* and *Na. asiatica* (which is also a specific feature in *Natrialbales*), while *Nn. gari* produced TGD instead. The absence of bis-sulfate glycosyl moieties and sulphated-Gly-AHH groups in *Nn. gari* further contributed to the differentiation of their lipidome. For phospholipids, highly abundant Gly-PG and PGS were exclusively found in *Nn. gari*. While minor cardiolipin of S-DGD-PA was present in *Nn. gari*, it represented the only detectable trace of cardiolipin within order *Natrialbales*.

The lipidome differentiation among the 7 strains grown under identical cultivation conditions was in alignment with their phylogenetic relationship at the order level. Such alignment underscores the mechanism of genetic control on lipid biosynthesis, uncovering the potential for a lipidomic approach in revealing microbial community dynamics in ancient environment when DNA, RNA or proteins were no longer available. However, this study only examined a limited number of strains and further investigations should expand into other archaeal clades.

## 5 Summary and conclusion

Our study provides a comprehensive view of the lipidome of Halobacteria. At the order level, the clustering results of both CLs and TLs are consistent with the phylogeny of Halobacteria, showing the potential of using lipids as biomarkers for phylogeny. In addition, some lipids are specific to certain taxa and can serve as diagnostic intact lipid biomarkers for specific groups within the Halobacteria, for example: TGD-PA in *Ha. argentinensis*, sulfate glycocardiolipins and abundant production of unsaturated archaeol in *Haloferax*, and bis-sulfate glycosyl lipids in *Natrialbales*. The integration of molecular network analysis in lipidomics expands the comprehensibility of lipidomic data and improves the accuracy of phylogenetic inference, aligning it more closely with the actual relationships among *Haloferax* species. Overall, our study highlights an application of lipidomics as a valuable tool for investigating archaeal phylogeny. Future research is warranted to deepen our understanding of the functional significance of lipidomics in Halobacteria and other archaeal lineages.

## Data availability statement

Publicly available datasets were analyzed in this study. This data can be found at: https://www.ncbi.nlm.nih.gov/nuccore/NR_176502.1, https://www.ncbi.nlm.nih.gov/nuccore/KT247970.1, https://www.ncbi.nlm.nih.gov/nuccore/NR_029142.2, https://www.ncbi.nlm.nih.gov/nuccore/NR_149760.1, https://www.ncbi.nlm.nih.gov/nuccore/NR_025555.1, https://www.ncbi.nlm.nih.gov/nuccore/NR_177325.1, https://www.ncbi.nlm.nih.gov/nuccore/NR_121590.1, https://www.ncbi.nlm.nih.gov/nuccore/NR_043389.1, https://www.ncbi.nlm.nih.gov/nuccore/NR_028244.1, https://www.ncbi.nlm.nih.gov/nuccore/NR_043803.1.

## Author contributions

WY: Conceptualization, Data curation, Formal analysis, Investigation, Methodology, Software, Visualization, Writing – original draft. WZ: Investigation, Methodology, Writing – review & editing. WH: Methodology, Writing – review & editing. WX: Funding acquisition, Supervision, Writing – review & editing. YC: Writing – review & editing. YZ: Supervision, Writing – review & editing. CZ: Conceptualization, Funding acquisition, Project administration, Resources, Supervision, Writing – review & editing. FZ: Methodology, Supervision, Project administration, Writing – review & editing, Conceptualization, Funding acquisition.

## Footnotes

^1^ https://metaboanalyst.ca
