# Supplementary material for: Lipidomic chemotaxonomy aligned with phylogeny of Halobacteria
Source: Front Microbiol. 2023 Nov 24;14:1297600. doi: 10.3389/fmicb.2023.1297600 (PMC10704169; doi:10.3389/fmicb.2023.1297600)
Supplement: Supplementary file 1 [file Table_1.docx]

| Strain | Accession number | Link |
| --- | --- | --- |
| Natrarchaeobaculum sulfurireducens AArc1 | NR_176502.1 | <https://www.ncbi.nlm.nih.gov/nuccore/NR_176502.1> |
| Natrarchaeobius chitinivorans AArcht-Bj | KT247970.1 | <https://www.ncbi.nlm.nih.gov/nuccore/KT247970.1> |
| Natronorubrum sulfidifaciens JCM 14089 | NR_029142.2 | <https://www.ncbi.nlm.nih.gov/nuccore/NR_029142.2> |
| Halodesulfurarchaeum formicicum HSR6 | NR_149760.1 | https://www.ncbi.nlm.nih.gov/nuccore/NR_149760.1 |
| Halobacterium salinarum 91-R6 | NR_025555.1 | <https://www.ncbi.nlm.nih.gov/nuccore/NR_025555.1> |
| Halococcoides cellulosivorans HArcel1 | NR_177325.1 | <https://www.ncbi.nlm.nih.gov/nuccore/NR_177325.1> |
| Haloarcula marismortui ATCC 43049 | NR_121590.1 | <https://www.ncbi.nlm.nih.gov/nuccore/NR_121590.1> |
| Halorubrum sodomense ATCC 33755 | NR_043389.1 | <https://www.ncbi.nlm.nih.gov/nuccore/NR_043389.1> |
| Halorubrum lacusprofundi ATCC 49239 | NR_028244.1 | <https://www.ncbi.nlm.nih.gov/nuccore/NR_028244.1> |
| Haloplanus natans RE 101 | NR_043803.1 | <https://www.ncbi.nlm.nih.gov/nuccore/NR_043803.1> |

**Table S1** Accession numbers and links of 10 reference 16S rRNA gene sequences from NCBI.

**Table S2** Detailed information of annotated lipids, including average retention time, metabolite name, formula, adduct type, theoretical m/z and detected m/z, and the mass error of the annotation.

| Lipid type | Lipid group | Metabolite name | Formula | Adduct type | Library Mz | Average Mz | Mass error (ppm) | S/N |
| --- | --- | --- | --- | --- | --- | --- | --- | --- |
| Archaeol | Archaeol | Archaeol (20:0_20:0) | C43H88O3 | [M+H]+ | 653.68 | 653.69 | 8.27 | 25431.95 |
| Archaeol | Archaeol | Archaeol (20:0_20:0) | C43H88O3 | [M+NH4]+ | 670.71 | 670.71 | 1.48 | 11322.29 |
| Archaeol | Archaeol | Archaeol (20:0_25:0) | C48H98O3 | [M+H]+ | 723.76 | 723.76 | 1.63 | 8155.11 |
| Archaeol | Archaeol | Archaeol (20:0_25:0) | C48H98O3 | [M+NH4]+ | 740.79 | 740.78 | 1.59 | 4058.09 |
| Archaeol | Archaeol | Archaeol (20:0_25:1) | C48H96O3 | [M+NH4]+ | 738.77 | 738.77 | 6.93 | 228.08 |
| Archaeol | Archaeol | Archaeol (20:0_25:2) | C48H94O3 | [M+NH4]+ | 736.75 | 736.76 | 2.20 | 103.88 |
| Archaeol | Archaeol | Archaeol (20:1_20:0) | C43H86O3 | [M+H]+ | 651.67 | 651.67 | 1.13 | 5764.58 |
| Archaeol | Archaeol | Archaeol (20:1_20:0) | C43H86O3 | [M+NH4]+ | 668.69 | 668.69 | 1.84 | 2385.31 |
| Archaeol | Archaeol | Archaeol (20:1_20:2) | C43H82O3 | [M+NH4]+ | 664.66 | 664.66 | 1.11 | 397.05 |
| Archaeol | Archaeol | Archaeol (20:1_20:3) | C43H80O3 | [M+H]+ | 645.62 | 645.62 | 1.58 | 158.98 |
| Archaeol | Archaeol | Archaeol (20:1_20:3) | C43H80O3 | [M+NH4]+ | 662.65 | 662.64 | 1.54 | 512.2 |
| Archaeol | Archaeol | Archaeol (20:1_25:4) | C48H88O3 | [M+NH4]+ | 730.71 | 730.71 | 2.94 | 485.21 |
| Archaeol | Archaeol | Archaeol (20:2_20:0) | C43H84O3 | [M+H]+ | 649.65 | 649.65 | 0.23 | 1557.49 |
| Archaeol | Archaeol | Archaeol (20:2_20:0) | C43H84O3 | [M+NH4]+ | 666.68 | 666.68 | 1.14 | 319.45 |
| Archaeol | Archaeol | Archaeol (20:2_20:3) | C43H78O3 | [M+NH4]+ | 660.63 | 660.63 | 0.76 | 415.49 |
| Archaeol | Archaeol | Archaeol (20:2_20:3) | C43H78O3 | [M+NH4]+ | 660.63 | 660.63 | 0.30 | 352.57 |
| Archaeol | Archaeol | Archaeol (20:2_25:0) | C48H94O3 | [M+NH4]+ | 736.75 | 736.75 | 1.96 | 455.51 |
| Archaeol | Archaeol | Archaeol (20:3_20:3) | C43H76O3 | [M+NH4]+ | 658.61 | 658.61 | 0.29 | 569.45 |
| Archaeol | Archaeol | Archaeol (20:3_25:0) | C48H92O3 | [M+NH4]+ | 734.74 | 734.74 | 0.73 | 792.46 |
| Archaeol | Archaeol | Archaeol (20:3_25:3) | C48H86O3 | [M+NH4]+ | 728.69 | 728.69 | 5.27 | 400.95 |
| Archaeol | Archaeol | Archaeol (20:3_25:4) | C48H84O3 | [M+NH4]+ | 726.68 | 726.67 | 2.73 | 974.82 |
| Archaeol | Archaeol | Archaeol (20:4_20:4) | C43H72O3 | [M+NH4]+ | 654.58 | 654.58 | 2.70 | 70.08 |
| Phospholipids | PA | PA (20:0_20:0) | C43H89O6P | [M+H]+ | 733.65 | 733.65 | 0.02 | 129.79 |
| Phospholipids | PE | PE (20:0_20:0) | C45H94O6NP | [M+H]+ | 776.69 | 776.69 | 0.56 | 1467.11 |
| Phospholipids | PE | PE (20:0_25:0) | C50H104O6NP | [M+H]+ | 846.77 | 846.77 | 3.67 | 180.74 |
| Phospholipids | Me-PGP | Me-PGP (20:0_20:0) | C47H98O11P2 | [M+H]+ | 901.67 | 901.67 | 9.47 | 31449.71 |
| Phospholipids | Me-PGP | Me-PGP (20:0_20:0) | C47H98O11P2 | [M+H]+ | 901.67 | 901.68 | 9.94 | 3597.79 |
| Phospholipids | Me-PGP | Me-PGP (20:0_25:0) | C52H108O11P2 | [M+H]+ | 971.74 | 971.75 | 5.20 | 25425.45 |
| Phospholipids | Me-PGP | Me-PGP (20:0_25:2) | C52H104O11P2 | [M+H]+ | 967.71 | 967.71 | 0.46 | 118.48 |
| Phospholipids | Me-PGP | Me-PGP (20:0_25:2) | C52H104O11P2 | [M+H]+ | 967.71 | 967.71 | 0.23 | 226.85 |
| Phospholipids | Me-PGP | Me-PGP (20:1_20:0) | C47H96O11P2 | [M+H]+ | 899.65 | 899.65 | 0.43 | 2037.87 |
| Phospholipids | Me-PGP | Me-PGP (20:1_20:2) | C47H92O11P2 | [M+H]+ | 895.62 | 895.62 | 0.19 | 414.77 |
| Phospholipids | Me-PGP | Me-PGP (20:1_20:2) | C47H92O11P2 | [M+H]+ | 895.62 | 895.62 | 1.65 | 477.64 |
| Phospholipids | Me-PGP | Me-PGP (20:1_25:0) | C52H106O11P2 | [M+H]+ | 969.73 | 969.73 | 0.67 | 573.13 |
| Phospholipids | Me-PGP | Me-PGP (20:2_20:0) | C47H94O11P2 | [M+H]+ | 897.63 | 897.64 | 2.22 | 1555.46 |
| Phospholipids | Me-PGP | Me-PGP (20:2_20:2) | C47H90O11P2 | [M+H]+ | 893.60 | 893.60 | 0.93 | 232.15 |
| Phospholipids | Me-PGP | Me-PGP (20:2_20:2) | C47H90O11P2 | [M+H]+ | 893.60 | 893.61 | 2.02 | 368.76 |
| Phospholipids | Me-PGP | Me-PGP (20:2_20:3) | C47H88O11P2 | [M+H]+ | 891.59 | 891.59 | 2.67 | 214.9 |
| Phospholipids | PG | PG (20:0_20:0) | C46H95O8P | [M+H]+ | 807.68 | 807.68 | 0.10 | 5731.43 |
| Phospholipids | PG | PG (20:0_20:0) | C46H95O8P | [M+H]+ | 807.68 | 807.69 | 2.02 | 1778.32 |
| Phospholipids | PG | PG (20:0_20:0) | C46H95O8P | [M+H]+ | 807.68 | 807.69 | 9.65 | 55860.47 |
| Phospholipids | PG | PG (20:0_20:0) | C46H95O8P | [M+NH4]+ | 824.71 | 824.71 | 0.84 | 160.37 |
| Phospholipids | PG | PG (20:0_25:0) | C51H105O8P | [M+H]+ | 877.76 | 877.76 | 1.76 | 1696.78 |
| Phospholipids | PG | PG (20:0_25:0) | C51H105O8P | [M+H]+ | 877.76 | 877.77 | 3.80 | 13104.62 |
| Phospholipids | PG | PG (20:0_25:0) | C51H105O8P | [M+NH4]+ | 894.79 | 894.79 | 0.91 | 73.1 |
| Phospholipids | PG | PG (20:0_25:1) | C51H103O8P | [M+H]+ | 875.75 | 875.75 | 1.25 | 242.92 |
| Phospholipids | PG | PG (20:0_25:2) | C51H101O8P | [M+H]+ | 873.73 | 873.73 | 2.20 | 225.5 |
| Phospholipids | PG | PG (20:1_20:0) | C46H93O8P | [M+H]+ | 805.67 | 805.67 | 0.54 | 4248.41 |
| Phospholipids | PG | PG (20:1_20:2) | C46H89O8P | [M+H]+ | 801.64 | 801.64 | 1.30 | 393.05 |
| Phospholipids | PG | PG (20:1_25:4) | C51H95O8P | [M+NH4]+ | 884.71 | 884.71 | 2.23 | 121.44 |
| Phospholipids | PG | PG (20:1_25:5) | C51H93O8P | [M+NH4]+ | 882.70 | 882.69 | 2.62 | 262.26 |
| Phospholipids | PG | PG (20:2_20:0) | C46H91O8P | [M+H]+ | 803.65 | 803.65 | 1.71 | 4154.4 |
| Phospholipids | PG | PG (20:2_20:2) | C46H87O8P | [M+H]+ | 799.62 | 799.62 | 3.25 | 387.06 |
| Phospholipids | PG | PG (20:2_20:3) | C46H85O8P | [M+H]+ | 797.61 | 797.60 | 2.16 | 106.98 |
| Phospholipids | PG | PG (20:3_20:3) | C46H83O8P | [M+H]+ | 795.59 | 795.59 | 1.02 | 376.58 |
| Phospholipids | PG | PG (20:3_20:3) | C46H83O8P | [M+NH4]+ | 812.62 | 812.62 | 1.14 | 290.18 |
| Phospholipids | PG | PG (20:3_25:4) | C51H91O8P | [M+NH4]+ | 880.68 | 880.68 | 0.73 | 122.56 |
| Phospholipids | PGS | PGS (20:0_20:0) | C46H95O11PS | [M+H]+ | 887.64 | 887.64 | 0.19 | 6621.69 |
| Phospholipids | PGS | PGS (20:0_20:0) | C46H95O11PS | [M+NH4]+ | 904.67 | 904.67 | 0.32 | 720.18 |
| Phospholipids | PGS | PGS (20:0_25:0) | C51H105O11PS | [M+H]+ | 957.72 | 957.72 | 0.44 | 2160.36 |
| Phospholipids | PGS | PGS (20:0_25:0) | C51H105O11PS | [M+NH4]+ | 974.75 | 974.75 | 0.80 | 243.53 |
| Phospholipids | PI | PI (20:0_20:0) | C49H99O11P | [M+H]+ | 895.70 | 895.70 | 2.26 | 221.4 |
| Phospholipids | Gly-PG | Gly-PG (20:0_20:0) | C52H105O13P | [M+H]+ | 969.74 | 969.74 | 0.64 | 2440.64 |
| Phospholipids | Gly-PG | Gly-PG (20:0_25:0) | C57H115O13P | [M+H]+ | 1039.82 | 1039.82 | 0.43 | 868.07 |
| Glycolipids | MGD | MGD (20:0_20:0) | C49H98O8 | [M+NH4]+ | 832.76 | 832.76 | 0.05 | 1026.1 |
| Glycolipids | MGD | MGD (20:0_20:0) | C49H98O8 | [M+NH4]+ | 832.76 | 832.76 | 0.54 | 104.97 |
| Glycolipids | MGD | MGD (20:0_20:0) | C49H98O8 | [M+NH4]+ | 832.76 | 832.76 | 2.29 | 1116.74 |
| Glycolipids | MGD | MGD (20:0_20:0) | C49H98O8 | [M+NH4]+ | 832.76 | 832.76 | 2.50 | 377.51 |
| Glycolipids | MGD | MGD (20:0_25:0) | C54H108O8 | [M+NH4]+ | 902.84 | 902.84 | 0.46 | 402.72 |
| Glycolipids | S-MGD | S-MGD (20:0_20:0) | C49H98O11S | [M+NH4]+ | 912.72 | 912.72 | 0.28 | 170.26 |
| Glycolipids | DGD | DGD (20:0_20:0) | C55H108O13 | [M+NH4]+ | 994.81 | 994.81 | 0.99 | 838.59 |
| Glycolipids | DGD | DGD (20:0_25:0) | C60H118O13 | [M+NH4]+ | 1064.89 | 1064.89 | 1.62 | 93.62 |
| Glycolipids | DGD | DGD (20:1_20:0) | C55H106O13 | [M+NH4]+ | 992.80 | 992.80 | 0.81 | 1349.29 |
| Glycolipids | DGD | DGD (20:2_20:0) | C55H104O13 | [M+NH4]+ | 990.78 | 990.78 | 1.44 | 374.76 |
| Glycolipids | DGD | DGD (20:2_20:2) | C55H100O13 | [M+NH4]+ | 986.75 | 986.75 | 0.34 | 161.75 |
| Glycolipids | S-DGD | S-DGD (20:0_20:0) | C55H108O16S | [M+NH4]+ | 1074.77 | 1074.77 | 0.64 | 2302.89 |
| Glycolipids | S-DGD | S-DGD (20:0_20:0) | C55H108O16S | [M+NH4]+ | 1074.77 | 1074.78 | 5.19 | 28830.26 |
| Glycolipids | S-DGD | S-DGD (20:0_25:0) | C60H118O16S | [M+NH4]+ | 1144.85 | 1144.85 | 1.21 | 405.47 |
| Glycolipids | S-DGD | S-DGD (20:0_25:0) | C60H118O16S | [M+NH4]+ | 1144.85 | 1144.85 | 1.24 | 1056.94 |
| Glycolipids | S-DGD | S-DGD (20:1_20:0) | C55H106O16S | [M+NH4]+ | 1072.75 | 1072.76 | 0.67 | 2042.39 |
| Glycolipids | S-DGD | S-DGD (20:1_20:2) | C55H102O16S | [M+NH4]+ | 1068.72 | 1068.72 | 3.28 | 112.08 |
| Glycolipids | S-DGD | S-DGD (20:2_20:0) | C55H104O16S | [M+NH4]+ | 1070.74 | 1070.74 | 1.13 | 1300.08 |
| Glycolipids | S-DGD | S-DGD (20:2_20:2) | C55H100O16S | [M+NH4]+ | 1066.71 | 1066.71 | 1.77 | 150.25 |
| Glycolipids | 2S-DGD | 2S-DGD (20:0_20:0) | C55H108O19S2 | [M+NH4]+ | 1154.73 | 1154.73 | 2.48 | 681.53 |
| Glycolipids | 2S-DGD | 2S-DGD (20:0_25:0) | C60H118O19S2 | [M+NH4]+ | 1224.81 | 1224.81 | 0.14 | 1514.98 |
| Glycolipids | TGD | TGD (20:0_20:0) | C61H118O18 | [M+H]+ | 1139.84 | 1139.84 | 1.24 | 74.84 |
| Glycolipids | TGD | TGD (20:0_20:0) | C61H118O18 | [M+H]+ | 1139.84 | 1139.84 | 1.36 | 2974.58 |
| Glycolipids | TGD | TGD (20:0_20:0) | C61H118O18 | [M+NH4]+ | 1156.87 | 1156.86 | 1.25 | 11768.16 |
| Glycolipids | TGD | TGD (20:0_20:0) | C61H118O18 | [M+NH4]+ | 1156.87 | 1156.87 | 2.02 | 10236.25 |
| Glycolipids | TGD | TGD (20:0_25:0) | C66H128O18 | [M+NH4]+ | 1226.94 | 1226.95 | 0.91 | 2786.71 |
| Glycolipids | TGD | TGD (20:1_20:0) | C61H116O18 | [M+NH4]+ | 1154.85 | 1154.85 | 1.01 | 75.33 |
| Glycolipids | S-Gly-AHH | S-Gly-AHH (20:0_20:0) | C55H111O16NS | [M+NH4]+ | 1091.80 | 1091.80 | 4.83 | 7387.49 |
| Glycolipids | S-Gly-AHH | S-Gly-AHH (20:0_25:0) | C60H121O16NS | [M+NH4]+ | 1161.87 | 1161.87 | 4.18 | 91.26 |
| Glycolipids | S-Gly-AHH | S-Gly-AHH (20:0_25:0) | C60H121O16NS | [M+NH4]+ | 1161.87 | 1161.87 | 1.88 | 136.07 |
| Glycolipids | S-Gly-AHH | S-Gly-AHH (20:1_20:0) | C55H109O16NS | [M+NH4]+ | 1089.78 | 1089.78 | 0.49 | 611.57 |
| Glycolipids | S-Gly-AHH | S-Gly-AHH (20:2_20:0) | C55H107O16NS | [M+NH4]+ | 1087.77 | 1087.76 | 0.95 | 276.26 |
| Glycolipids | S-Gly-AHH | S-Gly-AHH (20:2_20:0) | C55H107O16NS | [M+NH4]+ | 1087.77 | 1087.77 | 0.85 | 394.32 |
| Glycolipids | S-Gly-AHH | S-Gly-AHH (20:2_20:2) | C55H103O16NS | [M+NH4]+ | 1083.73 | 1083.73 | 0.56 | 94.7 |
| Glycolipids | S-Gly-AHH | S-Gly-AHH (20:3_20:0) | C55H105O16NS | [M+NH4]+ | 1085.75 | 1085.75 | 0.20 | 84.93 |
| Glycolipids | 2S-Gly-AHH | 2S-Gly-AHH (20:0_20:0) | C55H111O19NS2 | [M+NH4]+ | 1171.75 | 1171.76 | 1.35 | 799.87 |
| Glycolipids | 2S-Gly-AHH | 2S-Gly-AHH (20:0_25:0) | C60H121O19NS2 | [M+NH4]+ | 1241.83 | 1241.83 | 0.88 | 411.1 |
| Cardiolipin | BPG | BPG (20:0_20:0_20:0_20:0) | C89H182O13P2 | [M+H]+ | 1522.31 | 1522.32 | 1.09 | 490.92 |
| Cardiolipin | BPG | BPG (20:0_20:0_20:0_20:0) | C88TH182O13P2 | [M+H]+ | 1523.32 | 1523.31 | 1.67 | 66.61 |
| Cardiolipin | BPG | BPG (20:0_20:0_20:0_20:2) | C89H178O13P2 | [M+H]+ | 1518.28 | 1518.28 | 0.25 | 116.08 |
| Cardiolipin | BPG | BPG (20:0_20:0_20:0_20:3) | C89H176O13P2 | [M+H]+ | 1516.27 | 1516.27 | 0.50 | 97.4 |
| Cardiolipin | BPG | BPG (20:0_20:0_20:2_20:3) | C89H172O13P2 | [M+H]+ | 1512.24 | 1512.22 | 7.12 | 288.95 |
| Cardiolipin | BPG | BPG (20:0_20:0_20:2_20:3) | C89H172O13P2 | [M+H]+ | 1512.24 | 1512.23 | 3.26 | 259.79 |
| Cardiolipin | BPG | BPG (20:0_20:0_20:3_20:3) | C89H170O13P2 | [M+H]+ | 1510.22 | 1510.22 | 0.56 | 232.41 |
| Cardiolipin | BPG | BPG (20:0_20:0_20:3_20:3) | C88TH170O13P2 | [M+H]+ | 1511.22 | 1511.23 | 3.10 | 81.02 |
| Cardiolipin | BPG | BPG (20:0_20:2_20:2_20:3) | C89H168O13P2 | [M+H]+ | 1508.20 | 1508.20 | 1.20 | 92.88 |
| Cardiolipin | BPG | BPG (20:0_20:2_20:3_20:3) | C89H166O13P2 | [M+H]+ | 1506.19 | 1506.19 | 0.02 | 116.39 |
| Cardiolipin | MGD-PA | MGD-PA (20:0_20:0_20:0_20:0) | C92H185O13P | [M+H]+ | 1530.36 | 1530.36 | 1.24 | 242.42 |
| Cardiolipin | MGD-PA | MGD-PA (20:0_20:0_20:0_20:0) | C92H185O13P | [M+H]+ | 1530.36 | 1530.36 | 0.03 | 149.81 |
| Cardiolipin | DGD-PA | DGD-PA (20:0_20:0_20:0_20:0) | C98H195O18P | [M+H]+ | 1692.42 | 1692.42 | 0.02 | 1250.37 |
| Cardiolipin | DGD-PA | DGD-PA (20:0_20:0_20:0_20:0) | C98H195O18P | [M+H]+ | 1692.42 | 1692.42 | 0.63 | 281.38 |
| Cardiolipin | DGD-PA | DGD-PA (20:0_20:0_20:0_20:0) | C97TH195O18P | [M+NH4]+ | 1710.45 | 1710.37 | 45.62 | 84.81 |
| Cardiolipin | DGD-PA | DGD-PA (20:0_20:1_20:0_20:0) | C98H193O18P | [M+H]+ | 1690.40 | 1690.40 | 1.44 | 127.64 |
| Cardiolipin | DGD-PA | DGD-PA (20:0_20:2_20:0_20:0) | C98H191O18P | [M+H]+ | 1688.38 | 1688.38 | 0.57 | 80.47 |
| Cardiolipin | S-DGD-PA | S-DGD-PA (20:0_20:0_20:0_20:0) | C98H195O21PS | [M+H]+ | 1772.37 | 1772.37 | 2.86 | 134.41 |
| Cardiolipin | S-DGD-PA | S-DGD-PA (20:0_20:0_20:0_20:0) | C98H195O21PS | [M+H]+ | 1772.37 | 1772.37 | 0.31 | 2013.12 |
| Cardiolipin | S-DGD-PA | S-DGD-PA (20:0_20:0_20:0_20:0) | C97TH195O21PS | [M+H]+ | 1773.38 | 1773.38 | 0.49 | 95.49 |
| Cardiolipin | S-DGD-PA | S-DGD-PA (20:0_20:0_20:0_20:0) | C98H195O21PS | [M+NH4]+ | 1789.40 | 1789.40 | 0.34 | 4447.67 |
| Cardiolipin | S-DGD-PA | S-DGD-PA (20:0_20:0_20:0_20:0) | C97TH195O21PS | [M+NH4]+ | 1790.40 | 1790.40 | 0.99 | 6161.82 |
| Cardiolipin | S-DGD-PA | S-DGD-PA (20:0_20:0_20:0_20:2) | C98H191O21PS | [M+NH4]+ | 1785.37 | 1785.28 | 50.44 | 59.33 |
| Cardiolipin | S-DGD-PA | S-DGD-PA (20:0_20:0_20:0_20:3) | C98H189O21PS | [M+NH4]+ | 1783.35 | 1783.35 | 1.53 | 244.59 |
| Cardiolipin | S-DGD-PA | S-DGD-PA (20:0_20:0_20:0_20:3) | C97TH189O21PS | [M+NH4]+ | 1784.36 | 1784.28 | 39.87 | 54.71 |
| Cardiolipin | S-DGD-PA | S-DGD-PA (20:0_20:0_20:2_20:3) | C98H185O21PS | [M+NH4]+ | 1779.32 | 1779.31 | 4.67 | 322.97 |
| Cardiolipin | S-DGD-PA | S-DGD-PA (20:0_20:0_20:2_20:3) | C98H185O21PS | [M+NH4]+ | 1779.32 | 1779.31 | 3.71 | 297.82 |
| Cardiolipin | S-DGD-PA | S-DGD-PA (20:0_20:1_20:0_20:0) | C98H193O21PS | [M+H]+ | 1770.36 | 1770.36 | 1.01 | 210.02 |
| Cardiolipin | S-DGD-PA | S-DGD-PA (20:0_20:1_20:0_20:0) | C98H193O21PS | [M+NH4]+ | 1787.38 | 1787.39 | 1.45 | 541.5 |
| Cardiolipin | S-DGD-PA | S-DGD-PA (20:0_20:1_20:1_20:2) | C97TH187O21PS | [M+NH4]+ | 1782.34 | 1782.34 | 2.37 | 291.04 |
| Cardiolipin | S-DGD-PA | S-DGD-PA (20:0_20:1_20:1_20:4) | C98H183O21PS | [M+NH4]+ | 1777.31 | 1777.31 | 1.66 | 383.52 |
| Cardiolipin | S-DGD-PA | S-DGD-PA (20:0_20:1_20:1_20:4) | C97TH183O21PS | [M+NH4]+ | 1778.31 | 1778.31 | 2.86 | 481.15 |
| Cardiolipin | S-DGD-PA | S-DGD-PA (20:0_20:1_20:2_20:2) | C97TH185O21PS | [M+NH4]+ | 1780.32 | 1780.31 | 7.23 | 101.88 |
| Cardiolipin | S-DGD-PA | S-DGD-PA (20:0_20:2_20:0_20:0) | C98H191O21PS | [M+H]+ | 1768.34 | 1768.34 | 0.68 | 115.43 |
| Cardiolipin | S-DGD-PA | S-DGD-PA (20:0_20:2_20:0_20:0) | C98H191O21PS | [M+NH4]+ | 1785.37 | 1785.37 | 1.05 | 484.86 |
| Cardiolipin | S-DGD-PA | S-DGD-PA (20:0_20:4_20:0_20:0) | C98H187O21PS | [M+NH4]+ | 1781.34 | 1781.33 | 2.96 | 195.55 |
| Cardiolipin | TGD-PA | TGD-PA (20:0_20:0_20:0_20:0) | C104H205O23P | [M+H]+ | 1854.47 | 1854.47 | 0.00 | 3053.16 |
| Cardiolipin | TGD-PA | TGD-PA (20:0_20:0_20:0_20:0) | C103TH205O23P | [M+H]+ | 1855.47 | 1855.47 | 0.69 | 14560.74 |
| Cardiolipin | TGD-PA | TGD-PA (20:0_20:0_20:0_20:0) | C104H205O23P | [M+NH4]+ | 1871.50 | 1871.50 | 1.07 | 5695.11 |
| Cardiolipin | TGD-PA | TGD-PA (20:0_20:0_20:0_20:0) | C103TH205O23P | [M+NH4]+ | 1872.50 | 1872.50 | 0.78 | 7076.25 |
| Caroteinoid | Bacterioruberin | Bacterioruberin | C50H76O4 | [M+H]+ | 741.58 | 741.58 | 2.18 | 7252.06 |
| Caroteinoid | Bacterioruberin | Monoanhydrobacterioruberin | C50H74O3 | [M+H]+ | 723.57 | 723.57 | 1.48 | 102.27 |
| Caroteinoid | Bacterioruberin | Monoanhydrobacterioruberin | C50H74O3 | [M+H]+ | 723.57 | 723.57 | 1.56 | 1113.61 |
| Caroteinoid | Bacterioruberin | Bisanhydrobacterioruberin | C50H72O2 | [M+H]+ | 705.56 | 705.56 | 1.77 | 1256.08 |
| Caroteinoid | Bacterioruberin | Trisanhydrobacterioruberin | C50H70O | [M+H]+ | 687.55 | 687.55 | 0.51 | 325.70 |
| Quinone | MK | MK (4:2) | C31H44O2 | [M+H]+ | 449.34 | 449.31 | 76.15 | 1003.6 |
| Quinone | MK | MK (4:3) | C31H42O2 | [M+H]+ | 447.33 | 447.29 | 75.35 | 184.63 |
| Quinone | MK | MK (7:6) | C46H66O2 | [M+H]+ | 651.51 | 651.51 | 0.76 | 102.17 |
| Quinone | MK | MK (7:6) | C46H66O2 | [M+NH4]+ | 668.54 | 668.54 | 1.82 | 57.41 |
| Quinone | MK | MK (7:7) | C46H64O2 | [M+H]+ | 649.50 | 649.49 | 6.92 | 313.04 |
| Quinone | MK | MK (7:7) | C46H64O2 | [M+NH4]+ | 666.53 | 666.52 | 0.25 | 68.78 |
| Quinone | MK | MK (8:6) | C51H76O2 | [M+NH4]+ | 738.62 | 738.62 | 5.22 | 138.57 |
| Quinone | MK | MK (8:7) | C51H74O2 | [M+H]+ | 719.58 | 719.57 | 5.15 | 4968.84 |
| Quinone | MK | MK (8:7) | C51H74O2 | [M+H]+ | 719.58 | 719.58 | 0.23 | 761.06 |
| Quinone | MK | MK (8:7) | C51H74O2 | [M+NH4]+ | 736.60 | 736.60 | 2.71 | 5022.35 |
| Quinone | MK | MK (8:7) | C51H74O2 | [M+NH4]+ | 736.60 | 736.60 | 0.73 | 2200.18 |
| Quinone | MK | MK (8:8) | C51H72O2 | [M+H]+ | 717.56 | 717.56 | 4.29 | 241.79 |
| Quinone | MK | MK (8:8) | C51H72O2 | [M+H]+ | 717.56 | 717.56 | 4.05 | 22903.23 |
| Quinone | MK | MK (8:8) | C51H72O2 | [M+NH4]+ | 734.59 | 734.59 | 5.04 | 23083.4 |
| Quinone | MK | MK (9:9) | C56H80O2 | [M+H]+ | 785.62 | 785.62 | 0.24 | 68.93 |
| Quinone | MK | MK (9:9) | C56H80O2 | [M+NH4]+ | 802.65 | 802.65 | 4.34 | 96.2 |
