# Supplementary material for: Lipidomic chemotaxonomy aligned with phylogeny of Halobacteria
Source: Front Microbiol. 2023 Nov 24;14:1297600. doi: 10.3389/fmicb.2023.1297600 (PMC10704169; doi:10.3389/fmicb.2023.1297600)
Supplement: Supplementary file 2 [file Data_Sheet_1.docx]

**
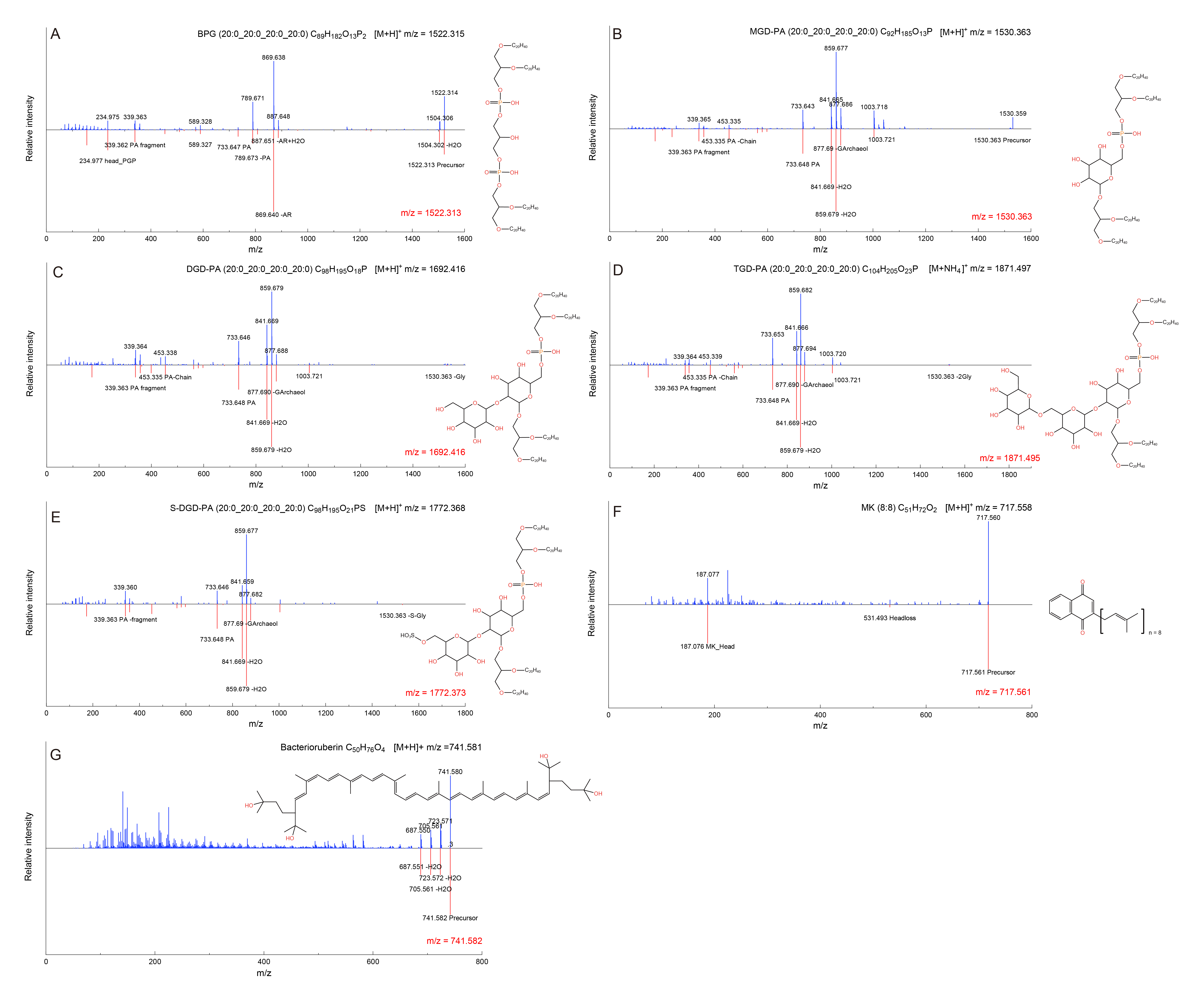
Figure S1** Mass spectra of annotated non-diether lipids and the corresponding spectra in the library. In each figure (A to H), the upper spectrum presents the MS^2^ detected from samples analyzed in this study and the lower one refers to the corresponding MS^2^ in the library. Precursor mass and adduct type are shown in the upper right corner of the figure. The m/z value in red color is the precursor mass in the library.

**
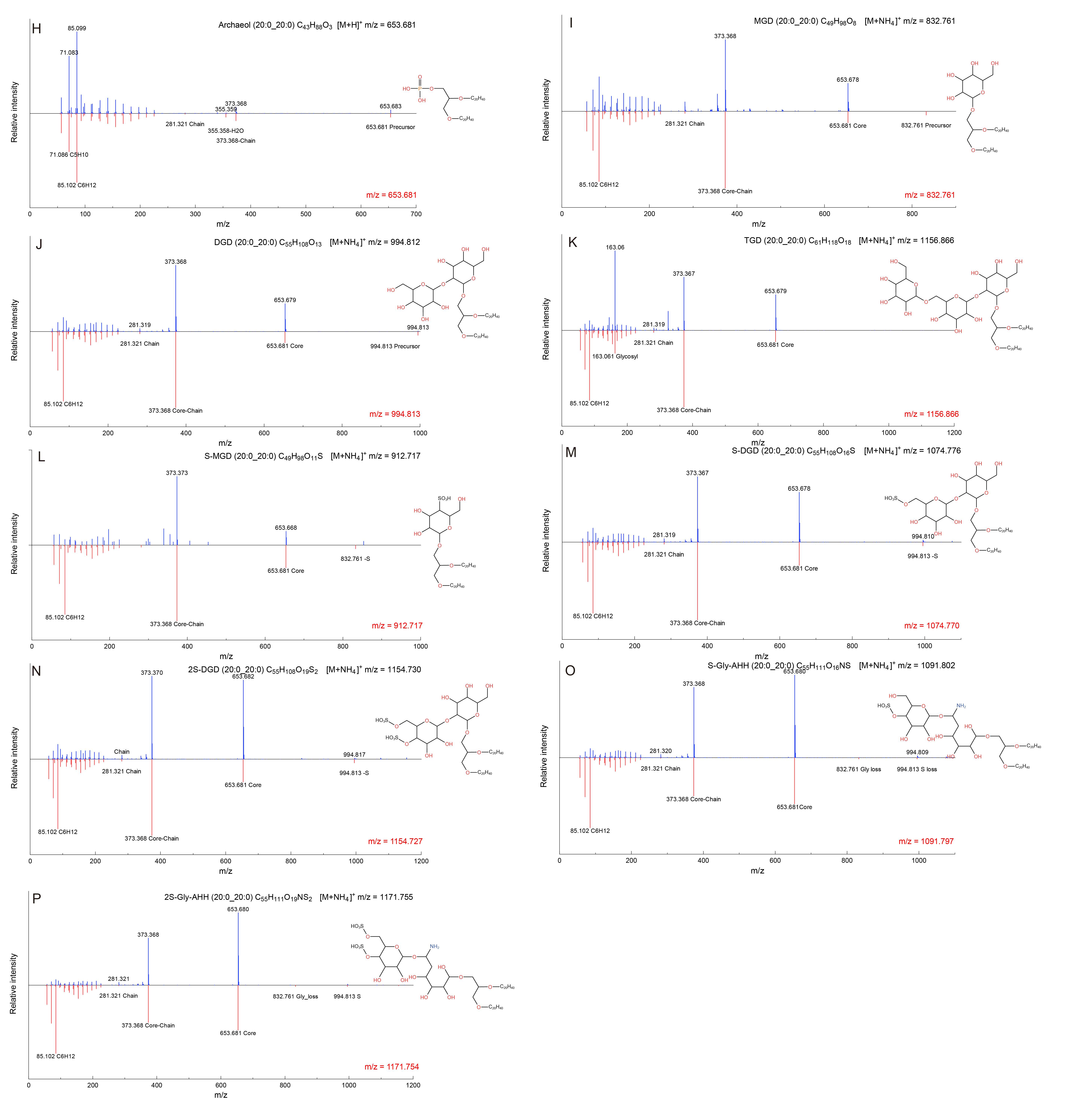
Figure S2** Mass spectra of annotated archaeol and glycolipids, and the corresponding spectra in the library. In each figure (I-Q), the upper spectrum presents the MS^2^ detected from samples analyzed in this study and the lower one refers to the corresponding MS^2^ in the library. Precursor mass and adduct type are shown in the upper right corner of the figure. The m/z value in red color is the precursor mass in the library.


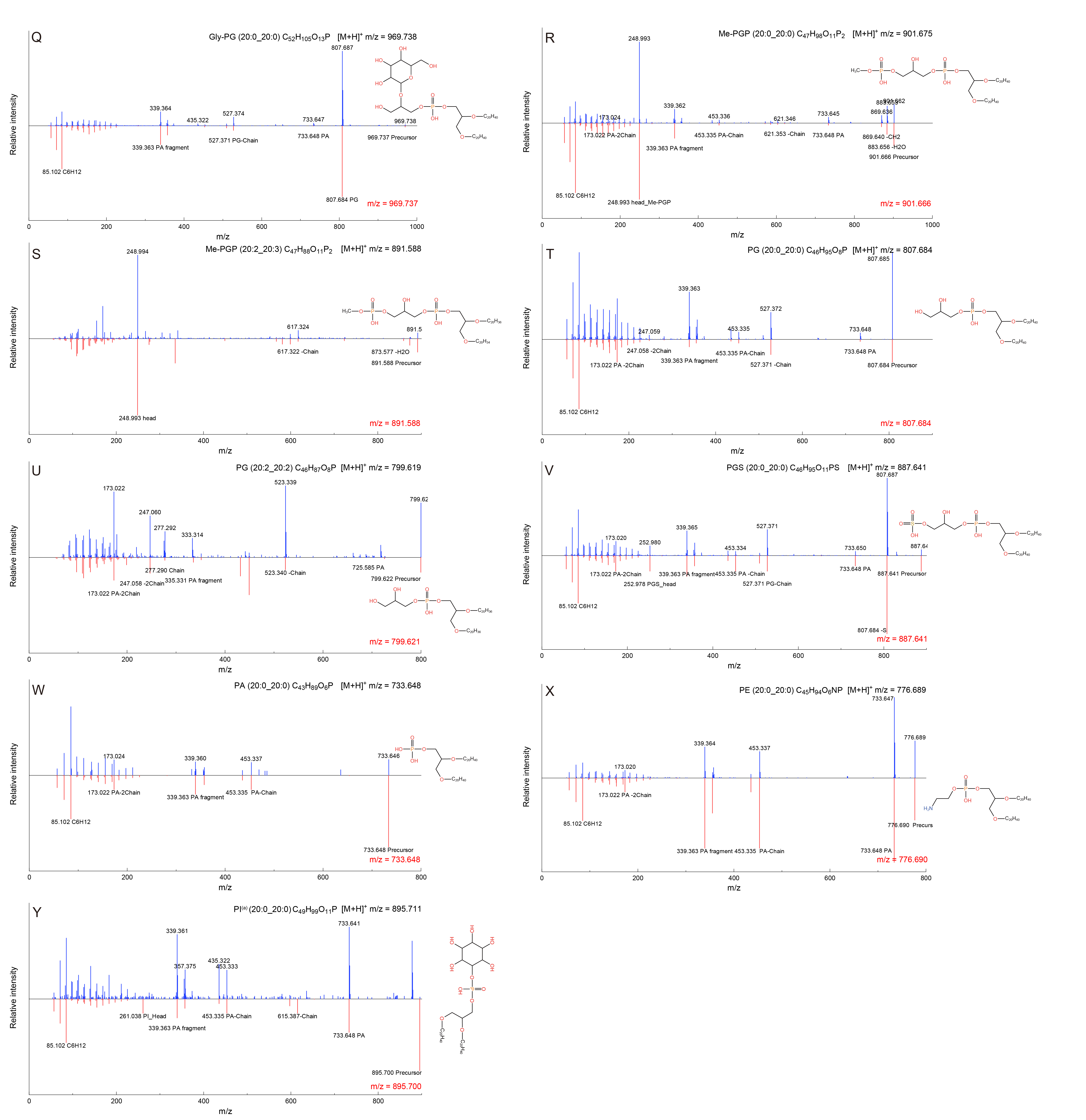


**Figure S3** Mass spectrum of annotated phospholipids and the corresponding spectrum in the library. In each figure (R-Z), the upper spectrum presents the MS^2^ detected from samples analyzed in this study and the lower one refers to the corresponding MS^2^ in the library. Precursor mass and adduct type are shown in the upper right corner of the figure. The m/z value in red color is the precursor mass in the library.

**
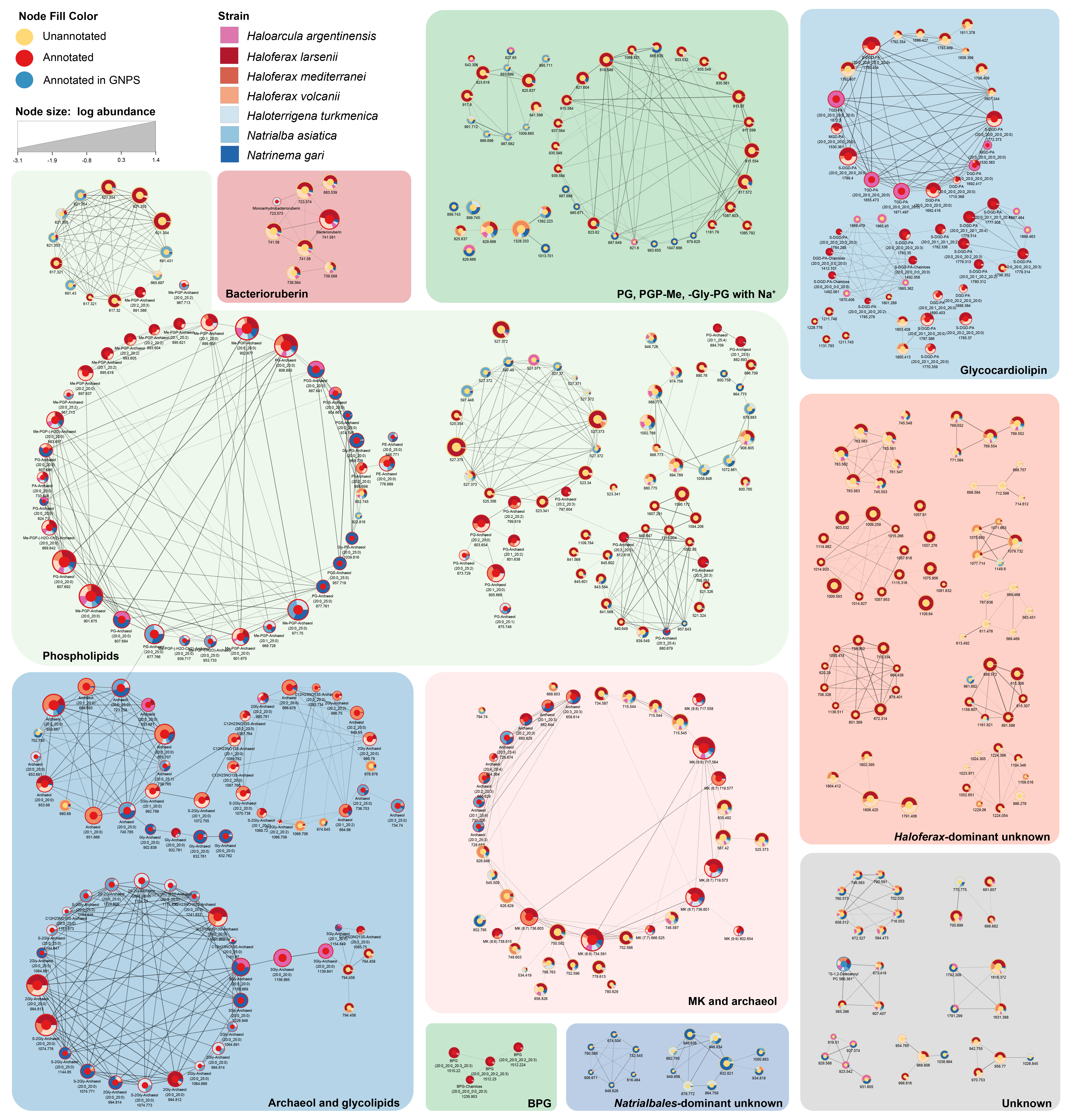
Figure S4** Feature-based molecular network of Halobacteria. The sub-networks are annotated based on the composition of the nodes and their occurrence in different strains. Edge thickness and opacity in the network represent the cosine score. Features with m/z < 500 and networks containing less than 4 nodes are filtered. In total, 444 features are involved in the networks. The annotated features are marked with a red core, while the unidentified lipids are with a yellow core.

**
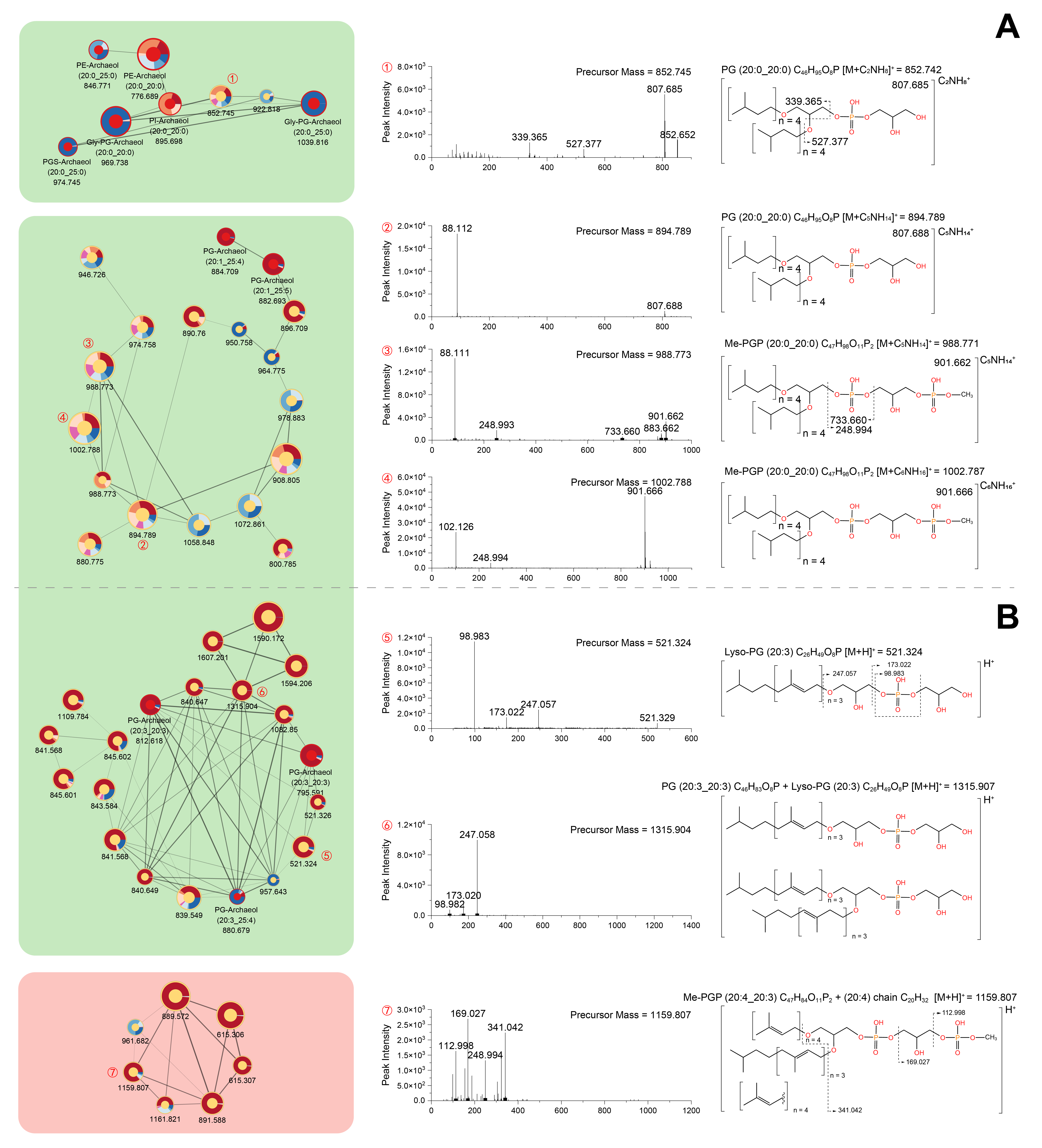
Figure S5** Network of the special form of PG and Me-PGP. A: Adduct forms of (CH_2_)_n_NH^3+^; B: Loss or addition of an unsaturated chain.

**
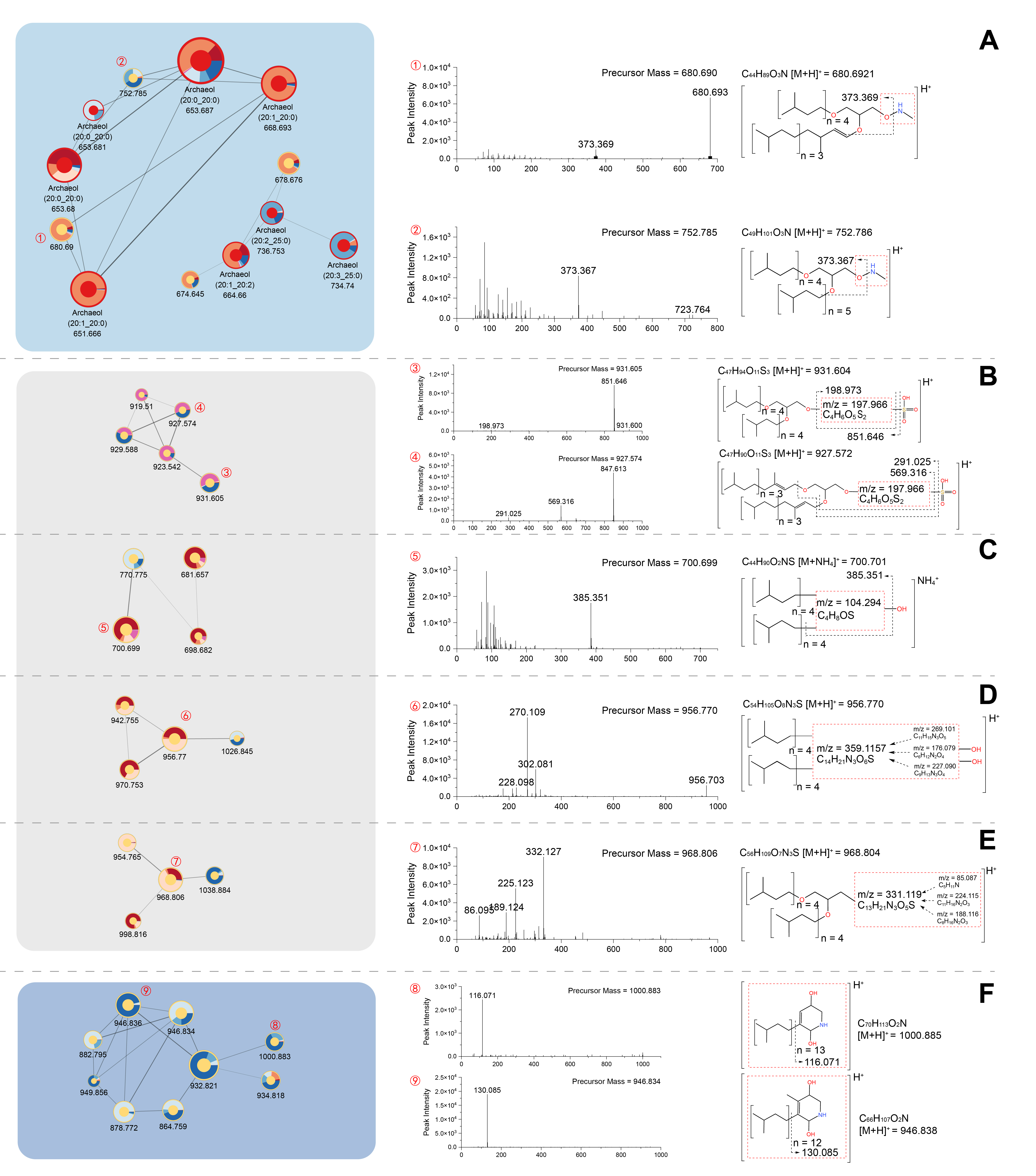
Figure S6** Network of the unknown structure containing valuable features. The molecular formula of the unknown structure is predicted in SIRUS. A ‒ E: diether-like network; F: quinone-like network.

**
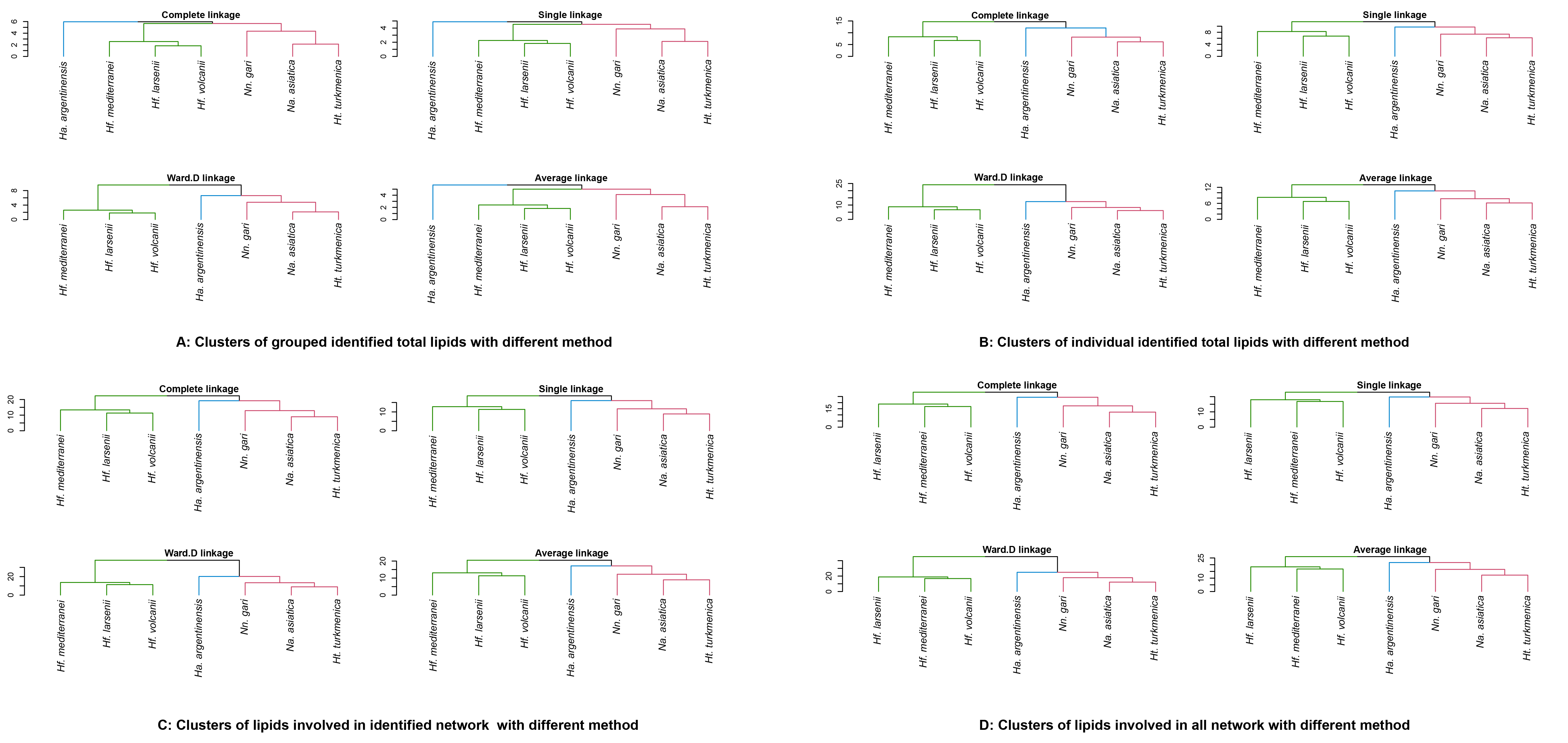
Figure S7** Cluster analysis with 4 different methods including complete linkage; single linkage; Ward. D linkage; average linkage. A: Clusters of grouped identified total lipids; B: Clusters of identical identified total lipids; C: Clusters of lipids in identified network; D: Clusters of lipids involved in all networks.

**
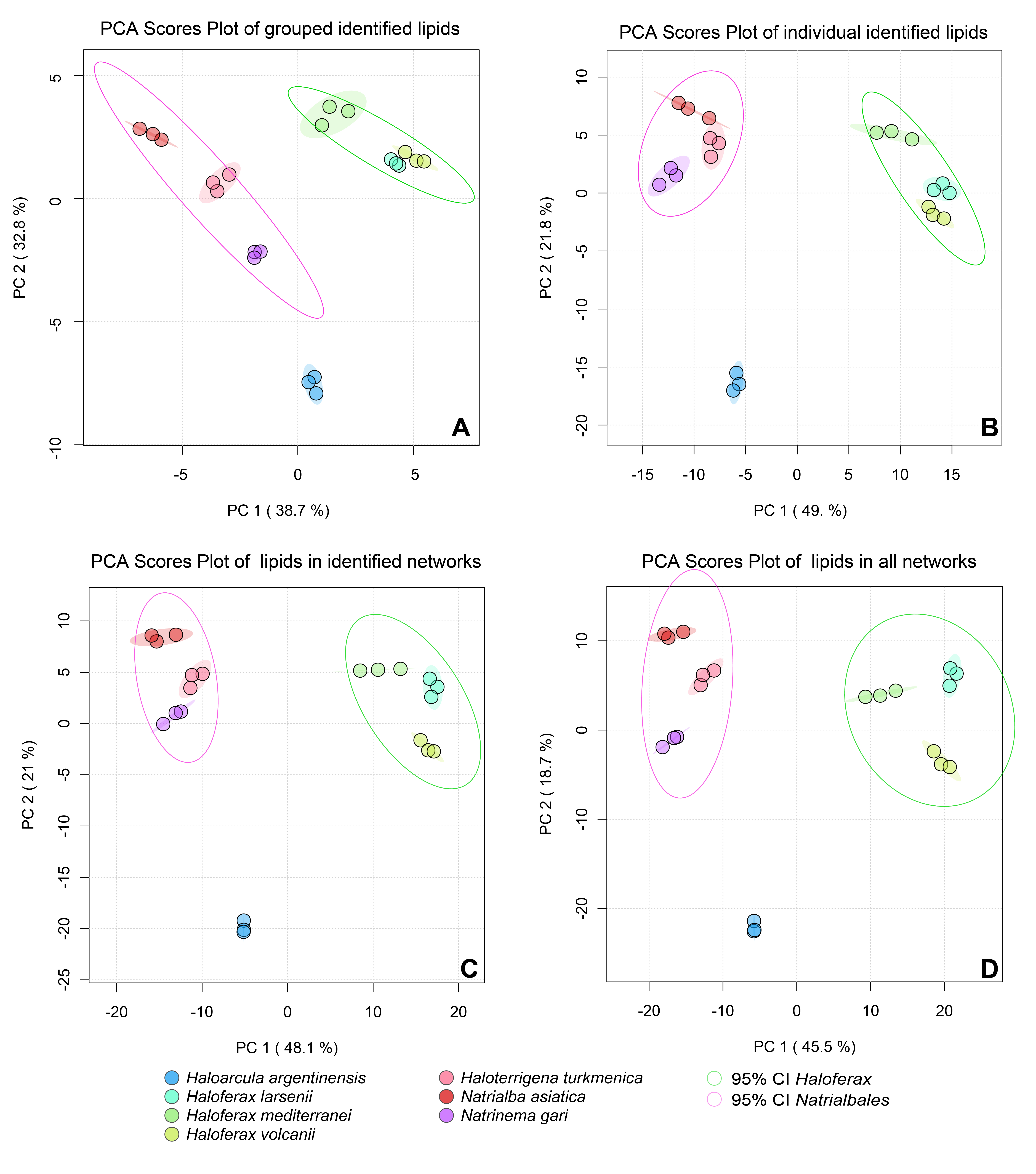
Figure S8** PCA score plot of strains using A) grouped annotated lipids, B) individual annotated lipids, C) lipids involved in identical networks, and D) lipids in all networks. Areas indicating 95% confidence interval (CI) for each strain or strain group are shown in solid sphere and empty sphere respectively.

**
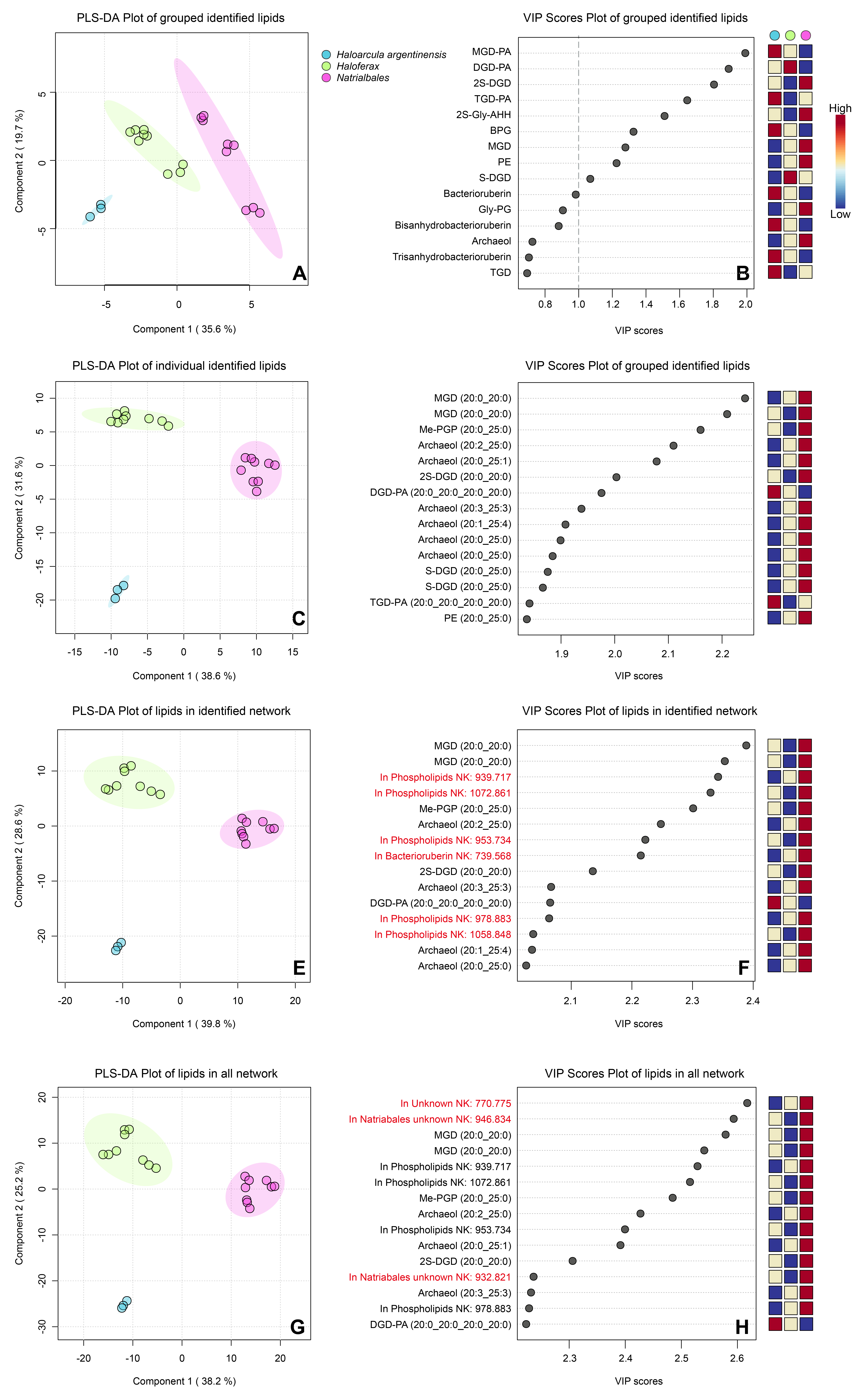
**

**Figure S9** PLS-DA plot of strains using A) grouped annotated lipids, C) individual annotated lipids, E) features involved in identified networks, and F) features in all networks. Areas indicating 95% confidence interval (CI) for strain group are shown in solid sphere. The corresponding VIP scores plot with top 15 features is shown in B, D, E and F. The dash line in B indicates VIP >1. Feature name shown in red indicate newly involved features in the analysis.
